# Ambipolar Charge Transport in Organic Semiconductors: How Intramolecular Reorganization Energy Is Controlled by Diradical Character

**DOI:** 10.3390/molecules28124642

**Published:** 2023-06-08

**Authors:** Yasi Dai, Andrea Zerbini, Juan Casado, Fabrizia Negri

**Affiliations:** 1 Department of Chemistry ‘Giacomo Ciamician’, Università di Bologna, Via F. Selmi, 2, 40126 Bologna, Italy; yasi.dai2@unibo.it (Y.D.); andrea.zerbini8@studio.unibo.it (A.Z.); 2 Department of Physical Chemistry, University of Málaga, Campus de Teatinos s/n, 29071 Málaga, Spain; casado@uma.es; 3 INSTM, UdR Bologna, Via F. Selmi, 2, 40126 Bologna, Italy

**Keywords:** DFT, diradical character, closed-shell, open-shell, conjugated diradicals, reorganization energy, charge transport, ambipolar transport, amphoteric behavior, broken symmetry orbitals

## Abstract

The charged forms of π–conjugated chromophores are relevant in the field of organic electronics as charge carriers in optoelectronic devices, but also as energy storage substrates in organic batteries. In this context, intramolecular reorganization energy plays an important role in controlling material efficiency. In this work, we investigate how the diradical character influences the reorganization energies of holes and electrons by considering a library of diradicaloid chromophores. We determine the reorganization energies with the four-point adiabatic potential method using quantum–chemical calculations at density functional theory (DFT) level. To assess the role of diradical character, we compare the results obtained, assuming both closed-shell and open-shell representations of the neutral species. The study shows how the diradical character impacts the geometrical and electronic structure of neutral species, which in turn control the magnitude of reorganization energies for both charge carriers. Based on computed geometries of neutral and charged species, we propose a simple scheme to rationalize the small, computed reorganization energies for both n-type and p-type charge transport. The study is supplemented with the calculation of intermolecular electronic couplings governing charge transport for selected diradicals, further supporting the ambipolar character of the investigated diradicals.

## 1. Introduction

π–conjugated chromophores are active components of optoelectronic devices [1,2,3,4,5,6] and energy storage substrates [7,8,9,10,11,12,13,14]. Both uses of organic semiconductors benefit from suitable redox properties since oxidation and reduction are correlated with the injection of holes into the highest occupied molecular orbital (HOMO) level and electrons into the lowest unoccupied molecular orbital (LUMO) level. Therefore, designing organic molecules with both redox properties (donor and acceptor abilities) is important to obtain novel ambipolar semiconductors that are of great interest due to the rapidly increasing demand for renewable energy. In this regard, to realize efficient ambipolar semiconductors, a relatively low band gap is desirable. This is a property that can be achieved exploiting not only with donor-acceptor polymers [15,16,17], but also with less common π–electron systems, such as antiaromatic compounds, radicals, and diradicals [18]. π–conjugated diradicals, thanks to their small HOMO–LUMO (H–L) gap, display absorption in the near infrared region, amphoteric electrochemical redox behaviour [19,20,21,22,23,24], and favourable p-type and n-type conduction. Such intrinsic electronic properties of organic semiconductors featuring unpaired electrons are, therefore, promising for advanced applications in optoelectronics, spintronics, and organic batteries [7,8].

Conjugated diradicals suffer from high reactivity, but significant efforts and recent protection strategies have contributed to design a large number of stable diradicals featuring an open-shell singlet ground state, different conjugated cores, and varying diradical character [20,25,26,27,28,29,30,31,32,33,34,35,36,37,38,39,40]. In parallel to the research committed to their chemical stabilization, there has been a widespread effort to rationalize the distinctive properties of diradicals from a theoretical point of view, including their linear and non-linear optical properties and their application in singlet fission processes [41,42,43,44,45,46,47].

Recently, diradicaloids displaying a balanced ambipolar carrier transport character with hole/electron carrier mobilities of the order of ca. 10^−3^ cm^2^V^−1^ s^−1^, and even higher, have been reported [20,21,23,48,49,50,51,52,53]. In most cases, the relatively low measured charge transport mobilities were ascribed to the introduction of bulky substituents, which are required to improve stability but detrimental for an effective molecular π–π packing and charge transport [20]. Several additional examples of OFETs based on diradicaloids have been reported, showing unipolar or unbalanced conduction [54,55,56,57,58,59,60,61,62,63,64].

The appearance of ambipolar behavior is influenced by a variety of factors. Efficient carrier injection from the electrodes must occur [65], but the ordered arrangement of molecules in the condensed phase and a reduction of grain boundaries [66] are also important to maximize the electronic couplings that govern charge transfer. In addition, the tuning of intra-molecular properties by chemical design and substitution is fundamental to optimize key parameters, such as electrode level alignment and intra-molecular reorganization energy. The latter plays an important role in material efficacy for optoelectronic and electrical devices, such as organic light emitting diodes (OLEDs) [67,68] and organic field-effect transistors (OFETs) [69,70,71]. By reference to the semiclassical Marcus theory, a small reorganization energy favors the inter-molecular charge hopping rate [72,73,74]. In the field of lighting materials, strategies to reduce the reorganization energy by innovative molecular designs have been the focus of recent studies [75]. Here, we discuss how reorganization energy is naturally reduced in diradicals thanks to their open-shell (OS) nature, and how the diradical character favors ambipolar conductance.

**Figure 1:**
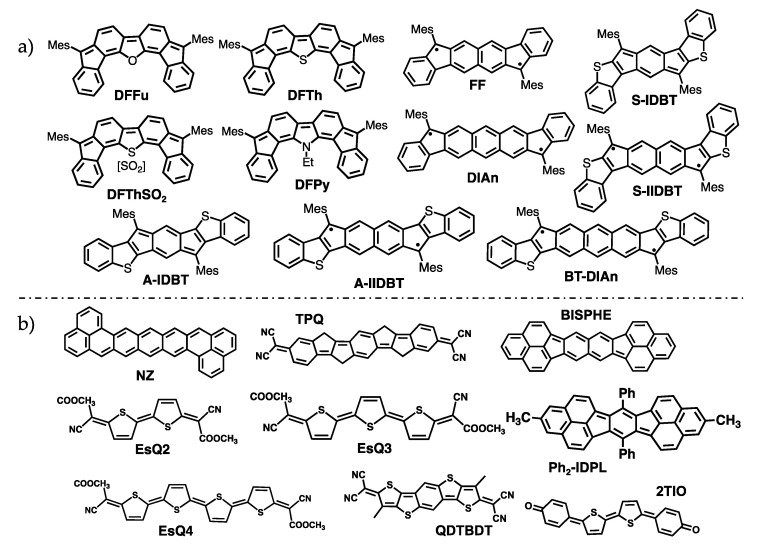
Structural formula of the conjugated diradicals (**a**) DFFu, DFTh, DFThSO_2_,DFPy, FF, DIAn, BT-DIAn, A/S-IDBT, and A/S-IIDBT, considered also in previous studies [49,64], and (**b**) NZ, TPQ, BISPHE, Ph_2_–IDPL, EsQn, QDTBDT, and 2TIO, whose intramolecular reorganization energies are investigated in this work for the first time.

In recent investigations on two classes of conjugated diradicals, difluorenoheterole compounds [49] and indaceno derivatives [64], we showed that the partial OS nature generates a pseudo-hole and pseudo-electron character in the neutral forms of these systems that, upon charging, provide similar conditions for the stabilization and transport of holes and electrons, resulting in amphoteric redox behavior and ambipolar conductance. Such behavior was shown to be assisted by rather small computed intramolecular reorganization energies. Here, we extend the study and consider an additional library of recently synthesized conjugated diradicals shown in the bottom part of Figure 1: NZ, 2TIO, QDTBDT, TPQ, EsQn, Ph_2_–IDPL, and BISPHE [22,26,33,47,48,76], some of which were previously investigated in the context of another distinctive character of conjugated diradicaloids, the appearance of a low-lying excited state featuring a doubly excited H,H→L,L orbital nature [46,47,77,78,79]. The entire set of diradicaloids is considered for the investigation of the relationship between intra-molecular reorganization energy, diradical character, and potential ambipolar electrical behavior. Based on the extended set of investigated diradicals, a simple two-dimensional representation of the PES of neutral and charged species is suggested to rationalize the reduced reorganization energies. The study is supplemented with the calculation of inter-molecular electronic couplings for selected diradicaloids, further supporting their propensity to ambipolar transport.

## 2. Results and Discussion

Quantum chemical calculations have been carried out to rationalize the connections between diradical character and ambipolar charge transport. In the framework of the non-adiabatic hopping model, the relevant charge-transfer event is localized on a molecular pair (dimer) formed by two neighboring molecules. A widely employed expression for the charge transfer rate constant is the Marcus equation [73,80]:

(1) $$k_{eT}=\frac{2\pi }{\hbar }V_{ij}{}^{2}\frac{1}{\sqrt{4\pi \lambda k_{B}T}}e^{\left(-\frac{{\left(\Delta G^{0}+\lambda \right)}^{2}}{4\lambda k_{B}T}\right)}$$

where *λ* is the intramolecular reorganization energy (neglecting external contributions to reorganization energies that are negligible in this context [81,82]), $V_{ij}$ are the intermolecular electronic couplings related with the through space overlap between the electronic wavefunctions of the two molecules forming the dimer, and $\Delta G^{0}$ is the driving force, which is zero for the self-exchange process of charge transfer between two identical molecules, in the absence of applied electric fields. Although the applicability of the hopping model is restricted to situations in which the coupling is smaller than the reorganization energy [83,84,85,86], it is clear that small reorganization energies favor charge mobilities.

Conjugated diradicaloids are OS systems, generally characterized by a singlet ground state due to double-spin polarization [87], and are easily represented as hybrids between quinoidal and biradical resonance structures. The contribution of each form in the ground state is described by the diradical character index ($y_{0}$), which is =0 for pure closed-shell (CS) systems and =1 for pure diradicals. From the computational point of view, such multiple resonance structures imply a significant contribution of static electron correlation, which is commonly introduced by the use of density functional theory (DFT) in its unrestricted formulation (UDFT). At this level of theory, the OS character implies broken symmetry (BS) molecular orbitals describing the localization of the unpaired electrons on opposite sides of the molecular backbone. In the following, we also label BS the correspondingly optimized geometries, as opposed to the CS label indicating molecular geometries optimized with the restricted DFT (RDFT) formulation.

To determine intramolecular reorganization energies λ, we investigated computationally neutral and charged species and used the four-point adiabatic potential method [88]. In addition to the BS description of the neutral species, we also determined the neutral CS ground state structures. While CS geometries for the neutral species are not sufficiently accurate for medium–large diradical character, they provide limiting reference values that can be compared with those obtained employing the more realistic BS geometries for the neutral compounds. Thus, a comparison between the description at CS and BS levels offers the key to understand how the diradical character influences the electronic structure and the molecular geometries, as well as how these control the intramolecular reorganization energies. In the following, we first assess how the diradical character impacts the electronic structure by correlating H/L gaps and orbital localizations with the diradical index $y_{0}$. Second, we show how the effect of orbital mixing determines the atomic structure of neutral diradical molecules. Finally, we discuss the computed trends in intra-molecular reorganization energies.

### 2.1. Frontier Orbital Energies, Diradical Character, Orbital Mixing

**Figure 2:**
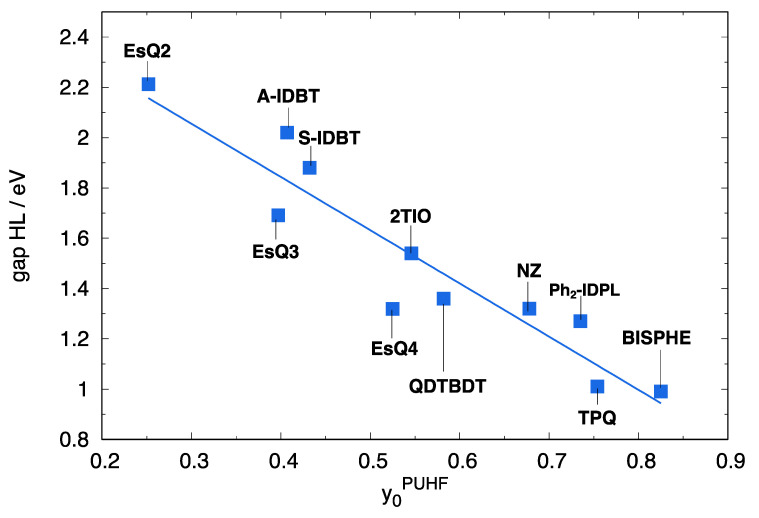
H/L gap computed at RB3LYP/6-311G* level for the library of diradicals, as a function of the computed diradical index $y_{0}^{PUHF}$. Calculations carried out at the CS optimized geometry.

Diradical molecules display a decreasing H/L gap with increasing diradical index $y_{0}$ (the latter computed at unrestricted Hartree Fock (UHF) level ($y_{0}^{PUHF}))$, as shown by the good linear dependence in Figure 2. Such decreasing H/L gap implies an increased role of static electron correlation and double-spin polarization effects that can be accounted for by determining the more stable BS geometries. The increased stabilization of the BS geometry, in a conjugated chromophore displaying a medium-to-large diradical character, is accompanied by an increased localization of the BS frontier orbitals driven by mixing CS frontier molecular orbitals [89]. Such mixing can thus be appreciated by expanding BS molecular orbitals as linear combinations of the CS counterparts, a procedure that we applied to the library of investigated molecules (Table S1).

**Figure 3:**
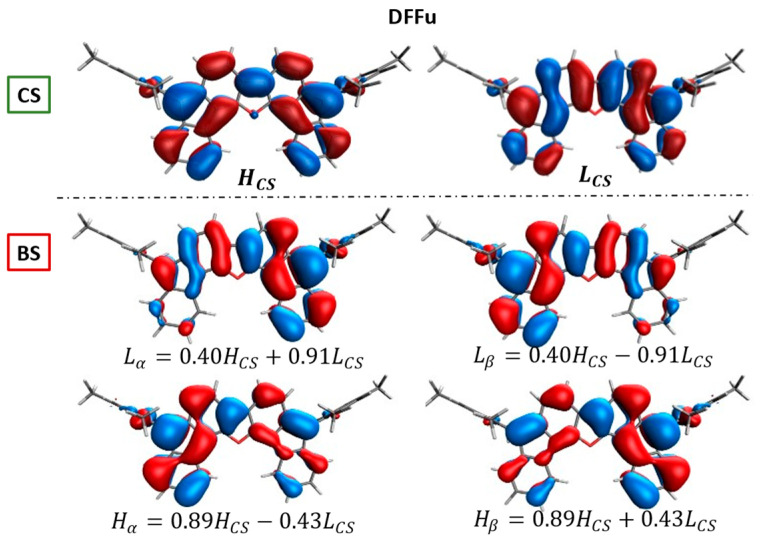
Comparison between CS (**top**) and BS (**bottom**) frontier molecular orbital shapes for neutral DFFu. CS and BS orbitals are obtained at the respectively optimized geometries, and the linear combinations of CS orbitals demonstrating the BS orbital mixing are also shown. From RB3LYP/6-311G* and UB3LYP/6-311G* calculations.

**Figure 4:**
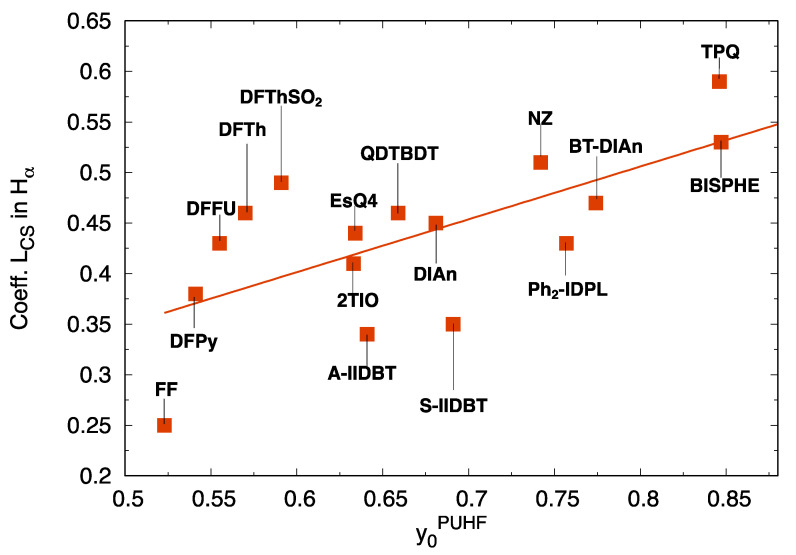
Contribution of the $L_{CS}$ to the $H_{\alpha }$ BS orbitals (from RB3LYP/6-311G* and UB3LYP/6-311G* calculations) as a function of the computed diradical index $y_{0}^{PUHF}$, for the library of investigated diradicals.

Such BS orbital localization and mixing is displayed, as an example, in Figure 3 for DFFu, where the linear combinations of CS orbitals, generating the BS orbitals, are also collected. As shown by the shape of BS-occupied orbitals and numerically demonstrated by the linear combination coefficients $H_{\alpha }$ and $H_{\beta }$, which contain a significant contribution (0.43) of the unoccupied $L_{CS}$ orbital. Mixing becomes larger, as expected, for larger diradical character (Table S1). This can be appreciated in Figure 4, where the contribution of the $L_{CS}$ to the $H_{\alpha }$ shows a general increase with the diradical index $y_{0}^{PUHF}$, accompanied by a similar decrease of the contribution of the $L_{CS}$ to the $L_{\alpha }$, as shown in Figure S1. 

**Figure 5:**
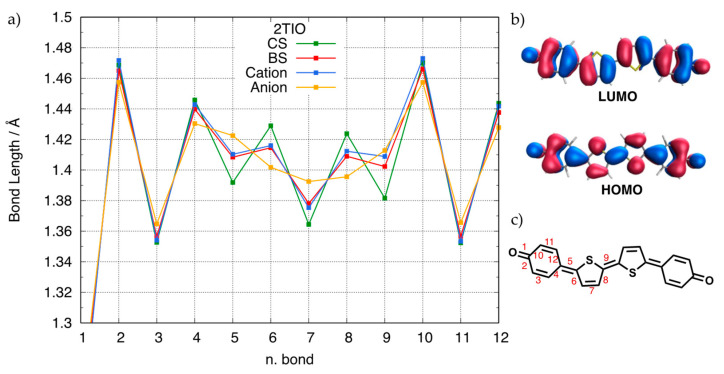
(**a**). Backbone bond lengths of 2TIO for the optimized geometries of the neutral CS (green), neutral BS (red), cation (blue), and anion (yellow) species (from RB3LYP/6-311G* and UB3LYP/6-311G* calculations). (**b**). Frontier CS molecular orbitals; (**c**). Numbering of selected skeleton bonds.

Note that orbital mixing crucially promotes the CS-to-BS geometry change, with the latter acquiring features consistent with the depletion of the $H_{CS}$ and concomitant occupation of the $L_{CS}$. For most conjugated diradicals, such a geometry change leads to a loss of quinoidal character and a recovery of aromaticity, which is exemplified in Figure 5 for 2TIO. The backbone bond lengths computed for the CS and BS geometries are depicted with green and red lines, respectively, together with those computed for the two charge carriers, namely the reduced and oxidized forms (yellow and blue lines, respectively). The BS neutral structure is characterized by a remarkably reduced quinoidal pattern, and the computed geometry change moves in the same direction of the geometries of charged species. Notably, both charged forms show remarkable deviations from the CS geometry (green) that can be rationalized by considering the density distribution of the orbital, from which the electron has been removed or to which the electron has been added. Specifically, the $H_{CS}$, also shown in Figure 5b, shows a density distribution that clearly reinforces a quinoidal geometry. Thus, removing one electron from such an orbital weakens the quinoidal structure and moves the geometry of the cation toward a more aromatic character. Similarly, the $L_{CS}$ displays a density distribution strongly favoring an aromaticity recovery, which is demonstrated by the change in bond length alternation along the central conjugation pattern of the anionic structure. In this specific case, the bond lengths of the neutral BS geometry are very similar to those of the cation. The above discussed orbital mixing occurring at UDFT level for the neutral species provides the explanation, since the partial depletion of the $H_{CS}$ in the BS-occupied molecular orbitals, causing a CS-to-BS geometry change that moves in the same direction of the formation of the cation. Thus, the exact geometry acquired by the neutral diradical depends on the magnitude of orbital mixing and can be tuned by the specific shape of the $H_{CS}/L_{CS}$ frontier orbitals.

### 2.2. Reorganization Energies from CS and BS Structures of the Neutral Species

**Figure 6:**
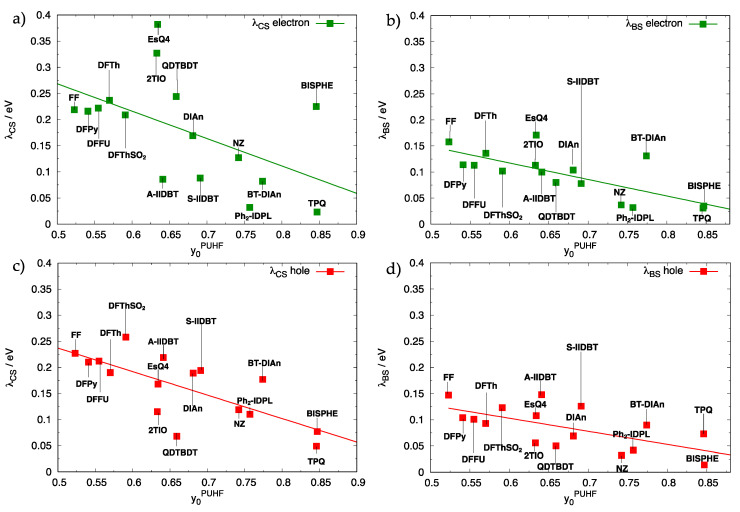
Intramolecular reorganization energies (in eV) computed for the formation of electron charge carriers (**a**,**b**) or hole charge carriers (**c**,**d**). Comparison between $\lambda_{CS}$ (**a**,**c**) computed using the CS geometry for the neutral species and $\lambda_{BS}$ (**b**,**d**) using the BS geometry.

The increased diradical character is reflected in a neutral BS structure consistent with the electron depletion of the $H_{CS}$ and filling of the $L_{CS}$, namely a geometry acquiring simultaneously a pseudo-hole and pseudo-electron character. Thus, the impact of diradical character can be assessed by comparing the computed λ by either assuming the CS potential energy surface (PES) of the neutral species (not including the contribution of diradical character), hereafter labelled $\lambda_{CS}$, or employing the BS PES (including the contribution of the diradical character) to obtain $\lambda_{BS}$. The calculations show that both sets of computed λ generally decrease for larger diradical character with a similar dependence on $y_{0}$ shown for hole and electron charge carriers (see Figure 6a–d and Table S2).

Notably, the use of CS structures leads, on average, to a two-fold increase of the computed $\lambda_{CS}$ (in some cases even more) compared to the $\lambda_{BS}$. Compare, for instance, the trend observed in Figure 6a,b for electron transport, which shows that the interpolated reorganization energy expected for a diradical character of ca. 0.5 is slightly larger than 0.25 eV, when the CS neutral geometry is adopted to evaluate λ and drops to ca. 0.15 eV for the BS geometry. Similarly, for hole charge transport (Figure 6c,d), the interpolated value for $y_{0}=0.5$ is slightly smaller than 0.25 eV using the neutral CS geometry, and it decreases to ca. 0.13 eV when the BS neutral structure is used to evaluate the reorganization energy.

**Figure 7:**
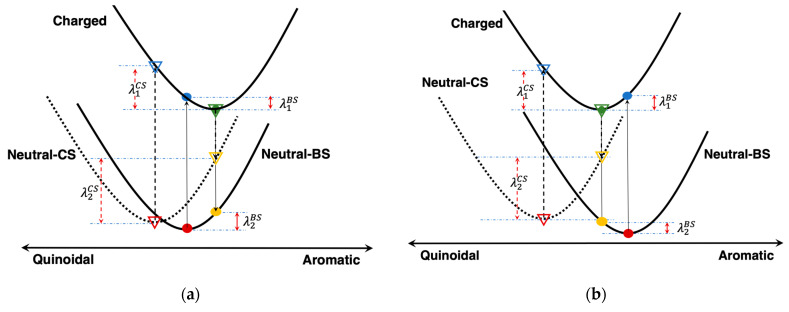
Schematic representation of how the diradical character and the consequent geometry change from CS-to-BS neutral structures impact the intramolecular reorganization energy: (**a**) Situation for small–medium diradical character: the PES of the BS structure is displaced toward more aromatic structures, but by a smaller amount compared to the charged species; (**b**) Situation for medium–large diradical character: the PES of the BS structure is displaced toward a more aromatic structure by a slightly larger amount compare to the charged species. In both cases, a decrease of the reorganization energy is expected with respect to the quinoidal CS structure. The color codes used for the four points required for the evaluation of the reorganization energy are: red = energy of the neutral species at its optimized geometry; yellow = energy of the neutral species at the geometry of the charged species; green = energy of the charged species at its optimized geometry; and blue = energy of the charged species at the geometry optimized for the neutral. Open triangles refer to points on the PES required for the calculation of $\lambda_{CS}$, while filled circles refer to points on the PES required for the calculation of $\lambda_{BS}$.

These trends reveal the preferential condition for charge transport of the BS diradical structure compared to the CS, a fact that can be easily rationalized by considering the simplified two-dimensional scheme shown in Figure 7. The two panels show the effect, promoted by the geometry change of the neutral species (when moving from the reference CS to the more realistic BS computed geometry), on the magnitude of reorganization energies. The scheme assumes that the geometries of the neutral (CS and BS) and charged species lay along a common nuclear displacement, bringing the molecular structure from fully quinoidal to fully aromatic. This is a reasonable assumption since the CS-to-BS geometry changes, as well as those induced by the redox processes, are determined by the nature of the same set of frontier molecular orbitals, H and L. Thus, the minima of the BS PES and those of the charged species will be found displaced toward more aromatic character. The magnitude of the displacement for the BS geometry depends on the diradical character: the larger the $y_{0}$, the larger the depletion of the $H_{CS}$ and concomitant increase of the $L_{CS}$ contribution, reinforcing aromaticity recovery. Thus, for a small–medium diradical character, the minimum of the BS neutral structure is likely to be displaced toward the geometry of the charged species, but it still keeps a certain quinoidal character. This corresponds to Figure 7a. For larger diradical characters, the minimum of the BS structure is energetically more stable and geometrically more displaced toward the aromatic structure, such that it may overcome the minimum of the charged species along the nuclear displacement coordinate, as shown in Figure 7b. In both cases, the reorganization energy, indicated in Figure 7 by the sum of the two contributions $\lambda_{1}^{BS}$ and $\lambda_{2}^{BS}$, is reduced compared to the sum of $\lambda_{1}^{CS}$ and $\lambda_{2}^{CS}$.

The overall remarkable result emerging for the library of diradicals is that the computed $\lambda_{BS}$ values have magnitudes around 0.1 eV, which are comparable with the best organic semiconductors, such as pentacene for hole transport [74]. Furthermore, the reorganization energy values are of the same magnitude for holes and electrons, which is a fundamental condition for ambipolar character. Clearly, ambipolar charge transport is crucially determined not only by small reorganization energies, but it also requires favorable level alignment and significant electronic couplings. Notably, in previous work, we showed that this latter condition is also satisfied in difluoreno–heteroles [49]. Here, we supplement the investigation of the intramolecular parameters by computing electronic couplings for two additional diradicals included in the library, whose crystal structures are available.

### 2.3. Electronic Couplings and Favorable Charge Transfer Paths

Vapour-deposited films of DIAn [20] and thin-films made by the diradical hydrocarbon with two phenalenyl radical moieties Ph_2_–IDPL displayed well-balanced ambipolar transport [21]. Both show small and substantially similar reorganization energies for n- and p-type conduction. To get further insight on their propensity to ambipolar conduction, we determined the most relevant charge transfer paths by inspecting the crystal of DIAn [20] and Ph_2_–IDPL [48] and evaluated the corresponding electronic couplings as outlined in Section 3.

**Figure 8:**
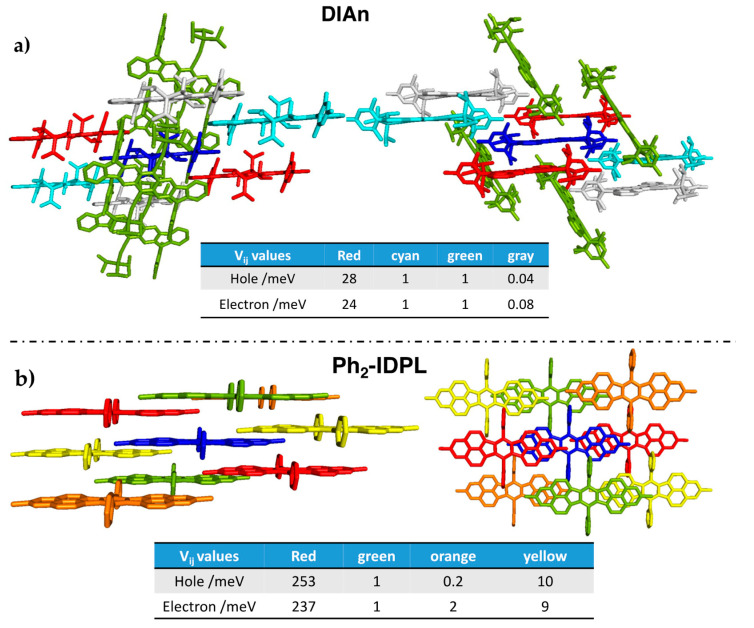
Selected cluster from the X-ray structure of (**a**) DIAn and (**b**) Ph_2_–IDPL corresponding to the most relevant paths for charge transport. Dimers are formed between the blue central molecule and the colored neighbors. Note that the same color indicates identical charge pathways. B3LYP/6-31G* computed electronic couplings, $V_{ij}$, are also reported.

In both cases, transport through the red channels shown in Figure 8a,b overcomes all the remaining pathways, as demonstrated by the largest calculated $V_{ij}$ values, clearly due to more efficient orbital overlap and shorter intermolecular distances. It should be noted that the interplanar distance in Ph_2_–IDPL is extremely reduced compared to most π–π stacked crystalline structures, which explains the huge computed $V_{ij}$ values in such case. However, the relevant point here is that electronic coupling values for hole and electron transport turned out to be rather similar, supporting similar transport mobilities for the two charge carrier types. Compared to the ideal crystal expectations, the reported experimental mobilities are reduced by the effective film structure in which molecular organization can be somewhat different from the crystal structure. Furthermore, additional morphological issues, such as grain size boundaries and defects, also contribute to reduced mobilities. Nevertheless, it is noteworthy that the combination of intra-molecular parameters such as reorganization energy and intermolecular electronic interactions concur to promote similar n-type and p-type transport efficiency.

## 3. Computational Methods

We performed gas–phase quantum chemical calculations in order to investigate electronic structures of the diradicals forming the library. Geometry optimization and frequency analysis for 2TIO, ESQ2, ESQ3, ESQ4, QDTBDT, NZ, TPQ, Ph_2_–IDPL, and BISPHE in the neutral singlet, and charged states were performed at the RB3LYP and UB3LYP levels, respectively, using the 6–311G* basis set. To obtain the BS wavefunctions, the “stable=opt” keyword was used. Specific procedures have been proposed to remove the spin contamination resulting from unrestricted level calculations [90,91] and to improve predicted energies of open-shell species. However, the calculation of reorganization energies required the determination of BS-optimized geometries, for which corrective schemes for energy gradients and Hessians are not available. Equilibrium structures of neutral and charged species of difluorenoheteroles [49], indenofluorene [92], and its larger congeners based on fluorenofluorene [93] (FF) and diindenoanthracene [94] (DIAn) derivatives were taken from previous investigations [49,64]. Quantum chemical calculations were performed with the Gaussian16 suite of programs [95].

The diradical descriptor $y_{0}$ was computed in the spin-unrestricted single-determinant formalism with the spin-projection scheme as [42,89,96]:

(2) $$y_{0}^{PUnrestricted}=1-\frac{2T_{0}}{1+T_{0}^{2}}$$

with $T_{0}$ calculated as:

(3) $$T_{0}=\frac{n_{HONO}-n_{LUNO}}{2}$$

and *n* is the occupation number of the frontier natural orbitals (NO). NO occupation numbers were determined at UHF level and, accordingly, the $y_{0}$ parameter is indicated as $y_{0}^{PUHF}$. Note that the value of the diradical index depends strongly on the level of theory (DFT, UHF) [45]. Thus, while all other molecular properties were evaluated at the DFT level, the $y_{0}$ was computed at UHF level since this is the reference method generally employed.

**Figure 9:**
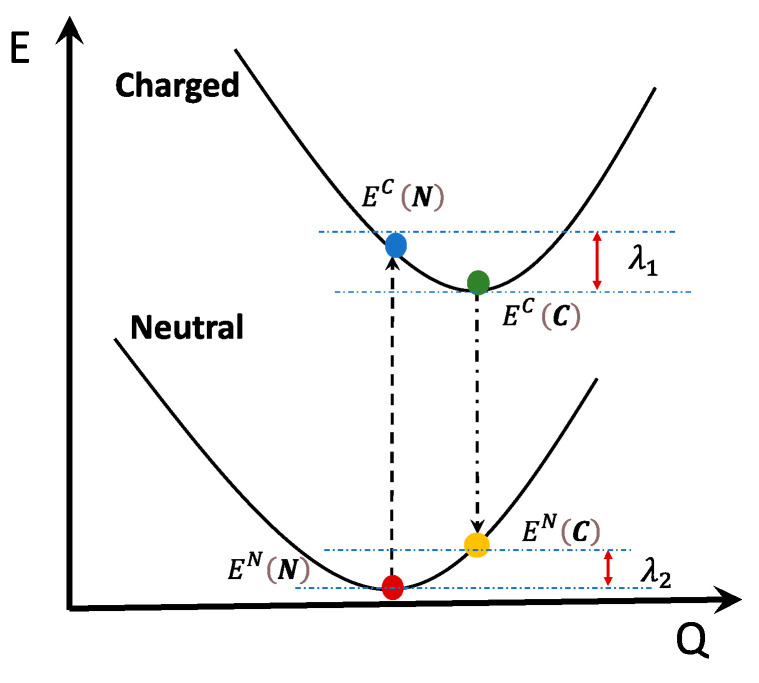
Schematic representation of the potential energy curves of neutral and charged species involved in the charge transfer process and indication of the two contributions $\lambda_{1}$ and $\lambda_{2}$ to the total intramolecular reorganization energy λ. The color codes used for the four points required to evaluate the reorganization energy are: red = energy of the neutral species at its optimized geometry; yellow: energy of the neutral species at the geometry of the charged species; green = energy of the charged species at its optimized geometry; and blue = energy of the charged species at the geometry optimized for the neutral.

The intramolecular reorganization energy λ was computed with the four-point adiabatic potential method, which is based on the evaluation of two energy values on the potential energy surface of neutral and charged states [72,88,97]. More precisely, the reorganization energy is the sum of two contributions (Figure 9): the first ($\lambda_{1}$) is computed as the difference between the energies of the charged species computed at the geometry of the neutral $E_{}^{C}\left(N\right)$ and at its optimized geometry $E_{}^{C}\left(C\right)$. The second contribution ($\lambda_{2}$) is determined as the difference between the energy of the neutral species at the equilibrium geometry of the charged $E_{}^{N}\left(C\right)$ and the energy calculated at its optimized geometry $E_{}^{N}\left(N\right)$. To assess the effect of increased diradical character, we determined two sets of reorganization parameters, either using the CS or BS neutral ground state geometries for all molecules displaying a lower energy BS structure.

To quantify the spin polarization effects in diradical species, we determined the changes between the set of BS molecular orbitals with respect to the CS counterparts. Such orbital mixing between BS and CS neutral structures was obtained by determining the linear combinations of CS orbitals that represent BS orbitals [89].

In the framework of the dimer approach and one-electron approximation, the intermolecular electronic coupling ($V_{ij}$ = $\langle \phi_{i}|\hat{H}|\phi_{j}\rangle$, where $\phi_{i}$ and $\phi_{j}$ are the highest occupied (HOMO) and lowest unoccupied molecular orbitals (LUMO), respectively, of the monomers forming the dimer) can be obtained with a fragment orbital approach, as reported in previous studies [98,99,100,101,102,103]. The electronic couplings were calculated at the crystalline geometries of the investigated systems [20,48], using the B3LYP functional and the 6-31G* basis set.

## 4. Conclusions

In this study, we focused on a specific class of organic semiconductors made by conjugated diradical molecules, and we explored the relationship between the diradical character and the magnitude of intramolecular reorganization energy, one of the relevant parameters governing charge transport but also influencing charging and discharging processes in batteries. More specifically, we investigated computationally the propensity to balanced p-type and n-type transport properties for a library of organic molecules characterized by varying diradical character.

Based on the comparison between CS and BS geometries of the neutral species, we showed how the localized electron pair in the latter promotes a geometry change in the same direction of the charged species. Such reduced geometry difference between neutral and charged species can be rationalized by simple electronic structure arguments, considering that the BS structure results from the mixing of $H_{CS}$ and $L_{CS}$ orbitals, that is, from the partial depletion of the occupied $H_{CS}$ and partial filling of the $L_{CS}$, similarly to the process occurring when charged species are generated. Thus, the diradical character imparts a degree of electron confinement that gives rise to the simultaneous generation of pseudo-hole and pseudo-electron character in the neutral species.

We showed that the magnitude of such BS geometry displacement in the direction of a more aromatic structure is more significant for larger diradical character and controls the decrease of reorganization energies for both types of charge carriers. More important, we found that the computed $\lambda_{BS}$ are comparable to those of best-performing organic semiconductors and display well-balanced values for hole and electron transport.

These trends for neutral and charged species suggested a simple two-dimensional representation of the PES of neutral and charged species accounting for the reduced reorganization energies, which we believe can serve as a guide to design more efficient ambipolar organic semiconductors based on diradicaloid chromophores.

The propensity to ambipolar conduction was further demonstrated by the similar values of computed electronic couplings for hole and electron transport of DIAn and Ph_2_-IDPL, two members of the library of diradicals whose ambipolarity was proved experimentally in previous studies [20,21].

Thus, this study sheds light on the impact of diradical character in determining low reorganization energies, which combined with optimal molecular packing and significant electronic couplings of similar magnitude for both charge carriers, making conjugated diradicals promising ambipolar semiconductors.

## Data Availability

Not applicable.

## Supplementary Materials

The following supporting information can be downloaded at: https://www.mdpi.com/article/10.3390/molecules28124642/s1; Figure S1: Contribution of the $L_{CS}$ to the $L_{\alpha }$ BS orbitals as a function of the computed diradical index $y_{0}^{PUHF}$ for the library of investigated diradicals; Table S1: Diradical characters and the linear combinations of HOMO and LUMO of the open-shell broken-symmetry (BS) structure for the library of investigated molecules; Table S2: Diradical characters and reorganization energies of the investigated library of diradicals.
