# Supplementary material for: Ambipolar Charge Transport in Organic Semiconductors: How Intramolecular Reorganization Energy Is Controlled by Diradical Character
Source: Molecules. 2023 Jun 8;28(12):4642. doi: 10.3390/molecules28124642 (PMC10304932; doi:10.3390/molecules28124642)
Supplement: Supplementary file 1 [file molecules-28-04642-s001.zip › molecules-2436254-supplementary.pdf]

Supplementary Materials for

Ambipolar Charge Transport in Organic Semiconductors: How Intramolecular Reorganization Energy Is Controlled by Diradical Character

Table S1. Diradical characters and the linear combinations of HOMO and LUMO of the open-shell broken-symmetry (BS) structure for the library of investigated molecules. BS orbitals are expressed as linear combination of closed-shell frontier molecular orbitals (*HCS* and *LCS*). Only the α orbitals are considered. Calculations from RB3LYP and UB3LYP levels of theory together with 6-311G* basis set. Diradical character are evaluated at PUHF/6-311G* level.

**Molecule**	y_0^{PUHF}	H_α		L_α	
		HCS	***LCS***	***HCS***	***LCS***
FF	0.523	0.90	0.25	-0.23	0.90
DFPy	0.541	0.92	-0.38	0.35	0.93
DFFU	0.555	0.89	-0.43	0.40	0.91
DFTh	0.570	0.88	-0.46	0.43	0.90
DFThSO2	0.591	0.86	-0.49	0.46	0.88
2TIO	0.633	-0.91	0.41	-0.39	-0.92
EsQ4	0.634	0.89	-0.44	0.43	0.90
A-IIDBT	0.641	-0.91	0.34	-0.32	-0.90
QDTBDT	0.659	0.88	-0.46	0.43	0.90
DIAn	0.681	0.85	0.45	0.42	-0.86
S-IIDBT	0.691	-0.91	0.35	0.33	0.92
NZ	0.742	0.86	-0.51	0.48	0.87
Ph2-IDPL	0.757	0.90	-0.43	-0.39	-0.92
BT-DIAn	0.774	0.87	-0.47	-0.46	-0.88
TPQ	0.846	0.76	-0.59	0.56	0.82
BISPHE	0.847	0.84	-0.53	-0.51	-0.86

Table S2. Diradical characters and reorganization energies of the investigated library of diradicals. Diradical characters are evaluated at the optimized geometries of the BS structures at PUHF/6-311G* level of theory. The reorganization energies are calculated both considering the CS (λ_{CS}) and the BS neutral PES (λ_{BS}) for hole and electron transports. Calculations carried out at RB3LYP and UB3LYP levels of theory with 6-311G* basis set.

**Molecule**	y_0^{PUHF} **@ geo BS**	λ_{CS}**(hole)**	λ_{CS}**(ele.)**	λ_{BS}**(hole)**	λ_{BS}**(ele.)**
EsQ2	-	0.201	0.489	-	-
EsQ3	-	0.183	0.429	-	-
A-IDBT	-	0.204	0.131	-	-
S-IDBT	-	0.285	0.197	-	-
FF	0.523	0.227	0.219	0.147	0.158
DFPy	0.541	0.210	0.216	0.104	0.114
DFFU	0.555	0.212	0.222	0.101	0.113
DFTh	0.570	0.190	0.237	0.093	0.136
DFThSO2	0.591	0.258	0.209	0.123	0.102
2TIO	0.633	0.115	0.327	0.056	0.113
EsQ4	0.634	0.168	0.382	0.108	0.171
A-IIDBT	0.641	0.219	0.086	0.148	0.100
QDTBDT	0.659	0.068	0.244	0.050	0.080
DIAn	0.681	0.189	0.169	0.069	0.104
S-IIDBT	0.691	0.194	0.088	0.126	0.078
NZ	0.742	0.119	0.127	0.032	0.037
Ph2-IDPL	0.757	0.110	0.032	0.042	0.032
BT-DIAn	0.774	0.177	0.082	0.090	0.131
TPQ	0.846	0.049	0.225	0.073	0.031
BISPHE	0.847	0.077	0.023	0.014	0.035

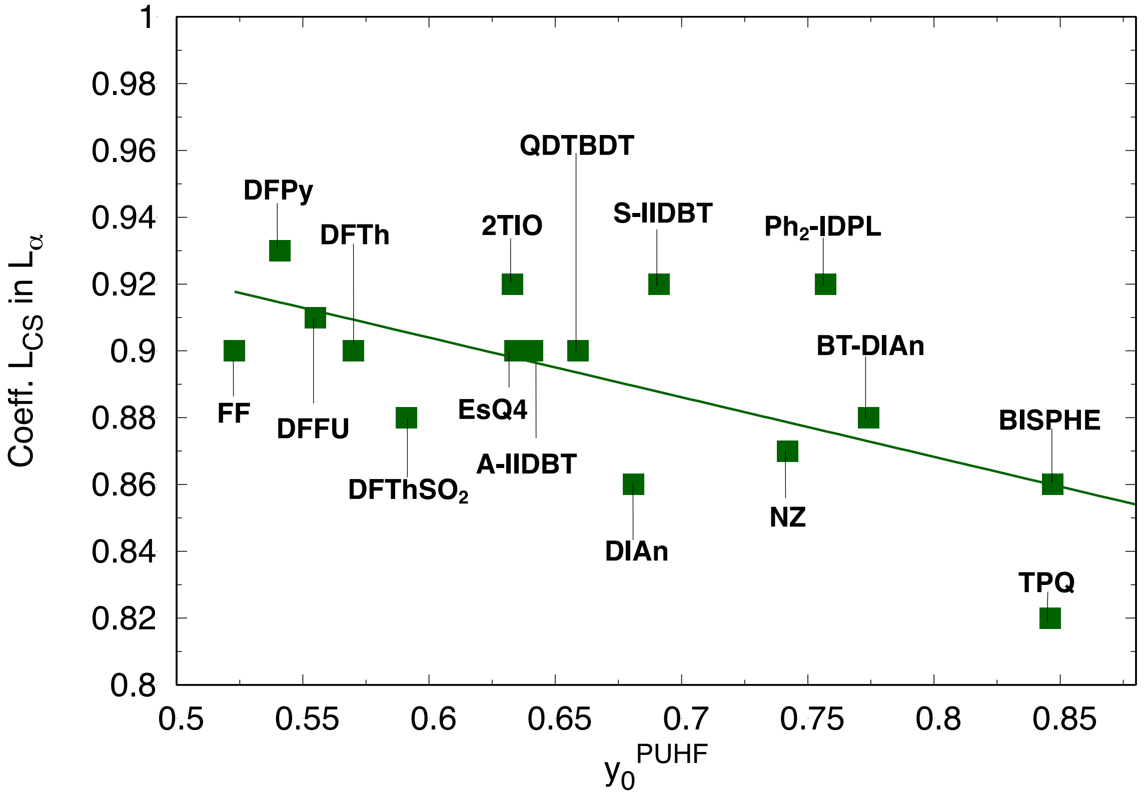

Figure S1. Contribution of the L_{CS} to the L_α BS orbitals (from RB3LYP/6-311G* and UB3LYP/6-311G* calculations), as a function of the computed diradical index y_0^{PUHF}, for the library of investigated diradicals.

OPTIMIZED GEOMETRIES

2TIO-Neutral: CS-B3LYP/6-311G* geometry

C	0.005386	0.690801	0.000000
C	-1.110131	1.575529	0.000000
C	-0.782625	2.900058	0.000000
C	0.620480	3.170901	0.000000
S	1.504122	1.633515	0.000000
H	-2.130484	1.211689	0.000000
H	-1.522274	3.688765	0.000000
C	-0.005386	-0.690801	0.000000
C	1.110131	-1.575529	0.000000
S	-1.504122	-1.633515	0.000000
C	0.782625	-2.900058	0.000000
H	2.130484	-1.211689	0.000000
C	-0.620480	-3.170901	0.000000
H	1.522274	-3.688765	0.000000
C	1.258635	4.407898	0.000000
C	2.697914	4.520721	0.000000
C	0.502695	5.640337	0.000000
C	3.322137	5.720406	0.000000
H	3.293225	3.612248	0.000000
C	1.110131	6.848933	0.000000
H	-0.580569	5.594522	0.000000
C	2.572385	6.984948	0.000000
H	4.403845	5.799023	0.000000
H	0.539045	7.771003	0.000000
C	-1.258635	-4.407898	0.000000
C	-0.502695	-5.640337	0.000000
C	-2.697914	-4.520721	0.000000
C	-1.110131	-6.848933	0.000000
H	0.580569	-5.594522	0.000000
C	-3.322137	-5.720406	0.000000
H	-3.293225	-3.612248	0.000000
C	-2.572385	-6.984948	0.000000
H	-0.539045	-7.771003	0.000000
H	-4.403845	-5.799023	0.000000
O	-3.132599	-8.078824	0.000000
O	3.132599	8.078824	0.000000

2TIO-Neutral: BS-UB3LYP/6-311G* geometry

C	-0.003198	0.701142	0.000000

C	-1.113571	1.568483	0.000000
C	-0.786940	2.907353	0.000000
C	0.602113	3.174733	0.000000
S	1.486793	1.643707	0.000000
H	-2.133193	1.202522	0.000000
H	-1.529890	3.693163	0.000000
C	0.003198	-0.701142	0.000000
C	1.113571	-1.568483	0.000000
S	-1.486793	-1.643707	0.000000
C	0.786940	-2.907353	0.000000
H	2.133193	-1.202522	0.000000
C	-0.602113	-3.174733	0.000000
H	1.529890	-3.693163	0.000000
C	1.253224	4.423597	0.000000
C	2.686950	4.528656	0.000000
C	0.501585	5.651587	0.000000
C	3.317516	5.729915	0.000000
H	3.280213	3.618900	0.000000
C	1.113571	6.862279	0.000000
H	-0.581822	5.608637	0.000000
C	2.572804	6.992664	0.000000
H	4.399626	5.803698	0.000000
H	0.544001	7.785390	0.000000
C	-1.253224	-4.423597	0.000000
C	-0.501585	-5.651587	0.000000
C	-2.686950	-4.528656	0.000000
C	-1.113571	-6.862279	0.000000
H	0.581822	-5.608637	0.000000
C	-3.317516	-5.729915	0.000000
H	-3.280213	-3.618900	0.000000
C	-2.572804	-6.992664	0.000000
H	-0.544001	-7.785390	0.000000
H	-4.399626	-5.803698	0.000000
O	-3.137541	-8.088678	0.000000
O	3.137541	8.088678	0.000000

2TIO-Cation: UB3LYP/6-311G* geometry

C	-0.000719	0.704466	0.000000
C	-1.118874	1.567187	0.000000
C	-0.789035	2.902474	0.000000
C	0.604072	3.156222	0.000000
S	1.483393	1.629719	0.000000
H	-2.139155	1.204452	0.000000
H	-1.529749	3.689842	0.000000
C	0.000719	-0.704466	0.000000

C	1.118874	-1.567187	0.000000
S	-1.483393	-1.629719	0.000000
C	0.789035	-2.902474	0.000000
H	2.139155	-1.204452	0.000000
C	-0.604072	-3.156222	0.000000
H	1.529749	-3.689842	0.000000
C	1.261956	4.403682	0.000000
C	2.701174	4.488789	0.000000
C	0.503246	5.630833	0.000000
C	3.337728	5.683188	0.000000
H	3.286352	3.574860	0.000000
C	1.118874	6.836866	0.000000
H	-0.578902	5.588535	0.000000
C	2.586235	6.950046	0.000000
H	4.419187	5.757732	0.000000
H	0.559145	7.765235	0.000000
C	-1.261956	-4.403682	0.000000
C	-0.503246	-5.630833	0.000000
C	-2.701174	-4.488789	0.000000
C	-1.118874	-6.836866	0.000000
H	0.578902	-5.588535	0.000000
C	-3.337728	-5.683188	0.000000
H	-3.286352	-3.574860	0.000000
C	-2.586235	-6.950046	0.000000
H	-0.559145	-7.765235	0.000000
H	-4.419187	-5.757732	0.000000
O	-3.152660	-8.035922	0.000000
O	3.152660	8.035922	0.000000

2TIO-Anion: UB3LYP/6-311G* geometry

C	0.007680	0.706374	0.000000
C	-1.065190	1.598947	0.000000
C	-0.701624	2.943183	0.000000
C	0.680008	3.180188	0.000000
S	1.522917	1.620685	0.000000
H	-2.095368	1.261343	0.000000
H	-1.427026	3.746501	0.000000
C	-0.007680	-0.706374	0.000000
C	1.065190	-1.598947	0.000000
S	-1.522917	-1.620685	0.000000
C	0.701624	-2.943183	0.000000
H	2.095368	-1.261343	0.000000
C	-0.680008	-3.180188	0.000000
H	1.427026	-3.746501	0.000000
C	1.379946	4.418576	0.000000

C	2.805879	4.490920	0.000000
C	0.680008	5.666066	0.000000
C	3.476257	5.680688	0.000000
H	3.377772	3.566034	0.000000
C	1.332192	6.864983	0.000000
H	-0.406127	5.661266	0.000000
C	2.786253	6.964231	0.000000
H	4.561899	5.712072	0.000000
H	0.781959	7.801580	0.000000
C	-1.379946	-4.418576	0.000000
C	-0.680008	-5.666066	0.000000
C	-2.805879	-4.490920	0.000000
C	-1.332192	-6.864983	0.000000
H	0.406127	-5.661266	0.000000
C	-3.476257	-5.680688	0.000000
H	-3.377772	-3.566034	0.000000
C	-2.786253	-6.964231	0.000000
H	-0.781959	-7.801580	0.000000
H	-4.561899	-5.712072	0.000000
O	-3.388319	-8.053958	0.000000
O	3.388319	8.053958	0.000000

EsQ2-Neutral: CS-B3LYP/6-311G* geometry

C	0.001327	0.685668	0.000000
C	1.144818	1.551244	0.000000
C	0.837620	2.871411	0.000000
C	-0.577569	3.147996	0.000000
S	-1.499964	1.647560	0.000000
H	2.158569	1.169440	0.000000
H	1.564145	3.673690	0.000000
C	-0.001327	-0.685668	0.000000
C	-1.144818	-1.551244	0.000000
S	1.499964	-1.647560	0.000000
C	-0.837620	-2.871411	0.000000
H	-2.158569	-1.169440	0.000000
C	0.577569	-3.147996	0.000000
H	-1.564145	-3.673690	0.000000
C	1.144818	-4.403705	0.000000
C	2.611207	-4.564090	0.000000
O	3.382918	-3.626393	0.000000
C	0.309177	-5.552361	0.000000
N	-0.400643	-6.465892	0.000000
O	2.985258	-5.849495	0.000000
C	4.403115	-6.094256	0.000000
H	4.505917	-7.175988	0.000000

H	4.862662	-5.662008	0.889216
H	4.862662	-5.662008	-0.889216
C	-1.144818	4.403705	0.000000
C	-0.309177	5.552361	0.000000
N	0.400643	6.465892	0.000000
C	-2.611207	4.564090	0.000000
O	-3.382918	3.626393	0.000000
O	-2.985258	5.849495	0.000000
C	-4.403115	6.094256	0.000000
H	-4.862662	5.662008	0.889216
H	-4.862662	5.662008	-0.889216
H	-4.505917	7.175988	0.000000

EsQ2-Cation: UB3LYP/6-311G* geometry

C	0.005070	0.702231	0.000000
C	1.154134	1.546164	0.000000
C	0.844613	2.874208	0.000000
C	-0.565342	3.133747	0.000000
S	-1.483056	1.629620	0.000000
H	2.167665	1.165168	0.000000
H	1.571890	3.676205	0.000000
C	-0.005070	-0.702231	0.000000
C	-1.154134	-1.546164	0.000000
S	1.483056	-1.629620	0.000000
C	-0.844613	-2.874208	0.000000
H	-2.167665	-1.165168	0.000000
C	0.565342	-3.133747	0.000000
H	-1.571890	-3.676205	0.000000
C	1.154134	-4.384179	0.000000
C	2.649111	-4.523943	0.000000
O	3.374347	-3.555324	0.000000
C	0.345625	-5.544875	0.000000
N	-0.339080	-6.478212	0.000000
O	3.021342	-5.788866	0.000000
C	4.449156	-6.054103	0.000000
H	4.532338	-7.135968	0.000000
H	4.902099	-5.624324	0.892289
H	4.902099	-5.624324	-0.892289
C	-1.154134	4.384179	0.000000
C	-0.345625	5.544875	0.000000
N	0.339080	6.478212	0.000000
C	-2.649111	4.523943	0.000000
O	-3.374347	3.555324	0.000000
O	-3.021342	5.788866	0.000000
C	-4.449156	6.054103	0.000000

H	-4.902099	5.624324	0.892289
H	-4.902099	5.624324	-0.892289
H	-4.532338	7.135968	0.000000

EsQ2-Anion: UB3LYP/6-311G* geometry

C	-0.013478	0.703875	0.000000
C	1.064512	1.598554	0.000000
C	0.696270	2.935662	0.000000
C	-0.696270	3.152860	0.000000
S	-1.544312	1.606277	0.000000
H	2.095648	1.264251	0.000000
H	1.397134	3.760888	0.000000
C	0.013478	-0.703875	0.000000
C	-1.064512	-1.598554	0.000000
S	1.544312	-1.606277	0.000000
C	-0.696270	-2.935662	0.000000
H	-2.095648	-1.264251	0.000000
C	0.696270	-3.152860	0.000000
H	-1.397134	-3.760888	0.000000
C	1.355856	-4.402230	0.000000
C	2.798585	-4.490445	0.000000
O	3.565339	-3.539318	0.000000
C	0.575300	-5.582158	0.000000
N	-0.099855	-6.526262	0.000000
O	3.250952	-5.779862	0.000000
C	4.668911	-5.925548	0.000000
H	4.851473	-6.999244	0.000000
H	5.111791	-5.465558	0.886305
H	5.111791	-5.465558	-0.886305
C	-1.355856	4.402230	0.000000
C	-0.575300	5.582158	0.000000
N	0.099855	6.526262	0.000000
C	-2.798585	4.490445	0.000000
O	-3.565339	3.539318	0.000000
O	-3.250952	5.779862	0.000000
C	-4.668911	5.925548	0.000000
H	-5.111791	5.465558	0.886305
H	-5.111791	5.465558	-0.886305
H	-4.851473	6.999244	0.000000

EsQ3-Neutral: CS-B3LYP/6-311G* geometry

C	0.000000	5.145672	0.573910
C	0.000000	4.569588	1.890830
C	0.000000	3.209952	1.902166

C	0.000000	2.616428	0.604335
S	0.000000	3.880418	-0.651430
H	0.000000	5.193326	2.775289
H	0.000000	2.618930	2.810417
C	0.000000	1.274708	0.292937
C	0.000000	0.680880	-1.007614
S	0.000000	-0.000000	1.525848
C	-0.000000	-0.680880	-1.007614
H	0.000000	1.281547	-1.908770
C	-0.000000	-1.274708	0.292937
H	-0.000000	-1.281547	-1.908770
C	-0.000000	-2.616428	0.604335
C	-0.000000	-3.209952	1.902166
S	-0.000000	-3.880418	-0.651430
C	-0.000000	-4.569588	1.890830
H	-0.000000	-2.618930	2.810417
C	-0.000000	-5.145672	0.573910
H	-0.000000	-5.193326	2.775289
C	0.000000	6.497385	0.287451
C	0.000000	7.437821	1.350703
N	0.000000	8.174661	2.243072
C	0.000000	6.965887	-1.107379
O	0.000000	6.215526	-2.063692
O	0.000000	8.303207	-1.201206
C	0.000000	8.840857	-2.534619
H	-0.888999	8.515764	-3.075950
H	0.000000	9.920163	-2.407585
H	0.888999	8.515764	-3.075950
C	-0.000000	-6.497385	0.287451
C	-0.000000	-7.437821	1.350703
N	-0.000000	-8.174661	2.243072
C	-0.000000	-6.965887	-1.107379
O	-0.000000	-6.215526	-2.063692
O	-0.000000	-8.303207	-1.201206
C	-0.000000	-8.840857	-2.534619
H	-0.000000	-9.920163	-2.407585
H	-0.888999	-8.515764	-3.075950
H	0.888999	-8.515764	-3.075950

EsQ3-Cation: UB3LYP/6-311G* geometry

C	0.000000	5.126937	0.589969
C	0.000000	4.545366	1.900489
C	0.000000	3.181551	1.894946
C	0.000000	2.621799	0.585832
S	0.000000	3.871131	-0.650470

H	0.000000	5.157661	2.793248
H	0.000000	2.578919	2.794745
C	0.000000	1.259263	0.251110
C	0.000000	0.687323	-1.042806
S	0.000000	-0.000000	1.473561
C	-0.000000	-0.687323	-1.042806
H	0.000000	1.285951	-1.945052
C	-0.000000	-1.259263	0.251110
H	-0.000000	-1.285951	-1.945052
C	-0.000000	-2.621799	0.585832
C	-0.000000	-3.181551	1.894946
S	-0.000000	-3.871131	-0.650470
C	-0.000000	-4.545366	1.900489
H	-0.000000	-2.578919	2.794745
C	-0.000000	-5.126937	0.589969
H	-0.000000	-5.157661	2.793248
C	0.000000	6.477378	0.308872
C	0.000000	7.418426	1.367802
N	0.000000	8.162552	2.253796
C	0.000000	6.957900	-1.106957
O	0.000000	6.188718	-2.042651
O	0.000000	8.278351	-1.181033
C	0.000000	8.856922	-2.509640
H	-0.891626	8.541623	-3.050119
H	0.000000	9.930227	-2.348475
H	0.891626	8.541623	-3.050119
C	-0.000000	-6.477378	0.308872
C	-0.000000	-7.418426	1.367802
N	-0.000000	-8.162552	2.253796
C	-0.000000	-6.957900	-1.106957
O	-0.000000	-6.188718	-2.042651
O	-0.000000	-8.278351	-1.181033
C	-0.000000	-8.856922	-2.509640
H	-0.000000	-9.930227	-2.348475
H	-0.891626	-8.541623	-3.050119
H	0.891626	-8.541623	-3.050119

EsQ3-Anion: UB3LYP/6-311G* geometry

C	-0.000000	5.181882	0.593383
C	-0.000000	4.605710	1.880925
C	-0.000000	3.219743	1.881940
C	-0.000000	2.642789	0.607363
S	-0.000000	3.914746	-0.632224
H	-0.000000	5.217072	2.774279
H	-0.000000	2.626507	2.789652

C	-0.000000	1.277586	0.258907
C	-0.000000	0.695497	-1.014524
S	-0.000000	-0.000000	1.485235
C	-0.000000	-0.695497	-1.014524
H	-0.000000	1.292191	-1.918786
C	-0.000000	-1.277586	0.258907
H	-0.000000	-1.292191	-1.918786
C	-0.000000	-2.642789	0.607363
C	-0.000000	-3.219743	1.881940
S	-0.000000	-3.914746	-0.632224
C	-0.000000	-4.605710	1.880925
H	-0.000000	-2.626507	2.789652
C	-0.000000	-5.181882	0.593383
H	-0.000000	-5.217072	2.774279
C	-0.000000	6.559003	0.286319
C	-0.000000	7.492609	1.349207
N	-0.000000	8.224346	2.249906
C	-0.000000	7.020144	-1.084682
O	-0.000000	6.297461	-2.069543
O	-0.000000	8.380410	-1.187062
C	-0.000000	8.890667	-2.519086
H	-0.886529	8.561497	-3.065527
H	-0.000000	9.974610	-2.415411
H	0.886529	8.561497	-3.065527
C	-0.000000	-6.559003	0.286319
C	-0.000000	-7.492609	1.349207
N	-0.000000	-8.224346	2.249906
C	-0.000000	-7.020144	-1.084682
O	-0.000000	-6.297461	-2.069543
O	-0.000000	-8.380410	-1.187062
C	-0.000000	-8.890667	-2.519086
H	-0.000000	-9.974610	-2.415411
H	-0.886529	-8.561497	-3.065527
H	0.886529	-8.561497	-3.065527

EsQ4-Neutral: CS-B3LYP/6-311G* geometry

C	0.705879	7.061770	0.000000
C	1.968386	6.380605	0.000000
C	1.867005	5.021930	0.000000
C	0.528236	4.538559	0.000000
S	-0.618439	5.900302	0.000000
H	2.901613	6.928634	0.000000
H	2.724255	4.358914	0.000000
C	0.102958	3.223779	0.000000
C	-1.237389	2.739971	0.000000

S	1.226017	1.852445	0.000000
C	-1.347409	1.377833	0.000000
H	-2.087317	3.411006	0.000000
C	-0.106090	0.684040	0.000000
H	-2.296304	0.855190	0.000000
C	0.106090	-0.684040	0.000000
C	1.347409	-1.377833	0.000000
S	-1.226017	-1.852445	0.000000
C	1.237389	-2.739971	0.000000
H	2.296304	-0.855190	0.000000
C	-0.102958	-3.223779	0.000000
H	2.087317	-3.411006	0.000000
C	-0.528236	-4.538559	0.000000
C	-1.867005	-5.021930	0.000000
S	0.618439	-5.900302	0.000000
C	-1.968386	-6.380605	0.000000
H	-2.724255	-4.358914	0.000000
C	-0.705879	-7.061770	0.000000
H	-2.901613	-6.928634	0.000000
C	-0.528236	-8.434548	0.000000
C	0.528236	8.434548	0.000000
C	-0.822292	9.013402	0.000000
O	-1.837868	8.344399	0.000000
O	-0.808518	10.355334	0.000000
C	-2.094218	10.997056	0.000000
H	-2.660356	10.716508	0.888846
H	-1.881993	12.062999	0.000000
H	-2.660356	10.716508	-0.888846
C	1.664132	9.284879	0.000000
N	2.614449	9.945731	0.000000
C	0.822292	-9.013402	0.000000
O	1.837868	-8.344399	0.000000
O	0.808518	-10.355334	0.000000
C	2.094218	-10.997056	0.000000
H	2.660356	-10.716508	0.888846
H	1.881993	-12.062999	0.000000
H	2.660356	-10.716508	-0.888846
C	-1.664132	-9.284879	0.000000
N	-2.614449	-9.945731	0.000000

EsQ4-Neutral: BS-UB3LYP/6-311G* geometry

C	0.704876	7.072148	0.000000
C	1.960009	6.394005	0.000000
C	1.854647	5.026668	0.000000
C	0.523213	4.553968	0.000000

S	-0.619203	5.909411	0.000000
H	2.895444	6.938268	0.000000
H	2.710858	4.362538	0.000000
C	0.081763	3.224699	0.000000
C	-1.243391	2.747675	0.000000
S	1.204400	1.860791	0.000000
C	-1.349917	1.369127	0.000000
H	-2.097233	3.413683	0.000000
C	-0.117845	0.694001	0.000000
H	-2.298032	0.845261	0.000000
C	0.117845	-0.694001	0.000000
C	1.349917	-1.369127	0.000000
S	-1.204400	-1.860791	0.000000
C	1.243391	-2.747675	0.000000
H	2.298032	-0.845261	0.000000
C	-0.081763	-3.224699	0.000000
H	2.097233	-3.413683	0.000000
C	-0.523213	-4.553968	0.000000
C	-1.854647	-5.026668	0.000000
S	0.619203	-5.909411	0.000000
C	-1.960009	-6.394005	0.000000
H	-2.710858	-4.362538	0.000000
C	-0.704876	-7.072148	0.000000
H	-2.895444	-6.938268	0.000000
C	-0.523213	-8.450263	0.000000
C	0.523213	8.450263	0.000000
C	-0.827615	9.030590	0.000000
O	-1.843882	8.363433	0.000000
O	-0.810823	10.372394	0.000000
C	-2.095374	11.016924	0.000000
H	-2.661924	10.737426	0.888890
H	-1.880795	12.082371	0.000000
H	-2.661924	10.737426	-0.888890
C	1.657256	9.300788	0.000000
N	2.607053	9.963074	0.000000
C	0.827615	-9.030590	0.000000
O	1.843882	-8.363433	0.000000
O	0.810823	-10.372394	0.000000
C	2.095374	-11.016924	0.000000
H	2.661924	-10.737426	0.888890
H	1.880795	-12.082371	0.000000
H	2.661924	-10.737426	-0.888890
C	-1.657256	-9.300788	0.000000
N	-2.607053	-9.963074	0.000000

EsQ4-Cation: UB3LYP/6-311G* geometry

C	0.695106	7.044387	0.000000
C	1.956607	6.362946	0.000000
C	1.847381	5.003297	0.000000
C	0.501249	4.541971	0.000000
S	-0.637137	5.885589	0.000000
H	2.893264	6.905573	0.000000
H	2.699236	4.334461	0.000000
C	0.064736	3.210540	0.000000
C	-1.263128	2.733230	0.000000
S	1.191456	1.857419	0.000000
C	-1.363310	1.358729	0.000000
H	-2.119953	3.395294	0.000000
C	-0.119782	0.694867	0.000000
H	-2.308337	0.830012	0.000000
C	0.119782	-0.694867	0.000000
C	1.363310	-1.358729	0.000000
S	-1.191456	-1.857419	0.000000
C	1.263128	-2.733230	0.000000
H	2.308337	-0.830012	0.000000
C	-0.064736	-3.210540	0.000000
H	2.119953	-3.395294	0.000000
C	-0.501249	-4.541971	0.000000
C	-1.847381	-5.003297	0.000000
S	0.637137	-5.885589	0.000000
C	-1.956607	-6.362946	0.000000
H	-2.699236	-4.334461	0.000000
C	-0.695106	-7.044387	0.000000
H	-2.893264	-6.905573	0.000000
C	-0.519478	-8.412278	0.000000
C	0.519478	8.412278	0.000000
C	-0.850724	8.999883	0.000000
O	-1.847381	8.310212	0.000000
O	-0.824361	10.325168	0.000000
C	-2.104965	10.998842	0.000000
H	-2.668765	10.725206	0.891155
H	-1.866202	12.057811	0.000000
H	-2.668765	10.725206	-0.891155
C	1.649826	9.267661	0.000000
N	2.592695	9.938070	0.000000
C	0.850724	-8.999883	0.000000
O	1.847381	-8.310212	0.000000
O	0.824361	-10.325168	0.000000
C	2.104965	-10.998842	0.000000
H	2.668765	-10.725206	0.891155
H	1.866202	-12.057811	0.000000
H	2.668765	-10.725206	-0.891155

C	-1.649826	-9.267661	0.000000
N	-2.592695	-9.938070	0.000000

EsQ4-Anion: UB3LYP/6-311G* geometry

C	0.717468	7.105114	0.000000
C	1.957113	6.430416	0.000000
C	1.849543	5.049160	0.000000
C	0.533949	4.574257	0.000000
S	-0.602994	5.937840	0.000000
H	2.895282	6.970339	0.000000
H	2.708578	4.387270	0.000000
C	0.078686	3.239782	0.000000
C	-1.236499	2.761310	0.000000
S	1.199619	1.870721	0.000000
C	-1.343797	1.372681	0.000000
H	-2.091192	3.426662	0.000000
C	-0.123180	0.696167	0.000000
H	-2.293432	0.850436	0.000000
C	0.123180	-0.696167	0.000000
C	1.343797	-1.372681	0.000000
S	-1.199619	-1.870721	0.000000
C	1.236499	-2.761310	0.000000
H	2.293432	-0.850436	0.000000
C	-0.078686	-3.239782	0.000000
H	2.091192	-3.426662	0.000000
C	-0.533949	-4.574257	0.000000
C	-1.849543	-5.049160	0.000000
S	0.602994	-5.937840	0.000000
C	-1.957113	-6.430416	0.000000
H	-2.708578	-4.387270	0.000000
C	-0.717468	-7.105114	0.000000
H	-2.895282	-6.970339	0.000000
C	-0.518831	-8.500861	0.000000
C	0.518831	8.500861	0.000000
C	-0.813630	9.066447	0.000000
O	-1.849543	8.419380	0.000000
O	-0.811271	10.429001	0.000000
C	-2.100269	11.041376	0.000000
H	-2.669982	10.755265	0.886678
H	-1.912540	12.113803	0.000000
H	-2.669982	10.755265	-0.886678
C	1.650819	9.349420	0.000000
N	2.605459	10.008970	0.000000
C	0.813630	-9.066447	0.000000
O	1.849543	-8.419380	0.000000

O	0.811271	-10.429001	0.000000
C	2.100269	-11.041376	0.000000
H	2.669982	-10.755265	0.886678
H	1.912540	-12.113803	0.000000
H	2.669982	-10.755265	-0.886678
C	-1.650819	-9.349420	0.000000
N	-2.605459	-10.008970	0.000000

QDTBDT-Neutral: CS-B3LYP/6-311G* geometry

C	0.124501	5.278037	0.000000
C	-1.199774	4.712430	0.000000
C	-1.129356	3.337904	0.000000
C	0.176456	2.774722	0.000000
S	1.391734	4.022302	0.000000
C	0.219646	1.390250	0.000000
C	-1.117845	0.831635	0.000000
S	-2.370958	2.091705	0.000000
C	1.343192	0.513479	0.000000
C	1.117845	-0.831635	0.000000
C	-0.219646	-1.390250	0.000000
C	-1.343192	-0.513479	0.000000
C	-0.176456	-2.774722	0.000000
C	1.129356	-3.337904	0.000000
S	2.370958	-2.091705	0.000000
C	1.199774	-4.712430	0.000000
C	-0.124501	-5.278037	0.000000
S	-1.391734	-4.022302	0.000000
C	2.477404	-5.498949	0.000000
C	-0.513652	-6.611209	0.000000
C	-1.893155	-6.954373	0.000000
N	-3.022144	-7.210028	0.000000
C	-2.477404	5.498949	0.000000
C	0.513652	6.611209	0.000000
C	1.893155	6.954373	0.000000
N	3.022144	7.210028	0.000000
C	-0.400531	7.697600	0.000000
N	-1.117845	8.605911	0.000000
C	0.400531	-7.697600	0.000000
N	1.117845	-8.605911	0.000000
H	2.348415	0.920476	0.000000
H	-2.348415	-0.920476	0.000000
H	2.557777	-6.144813	0.877224
H	2.557777	-6.144813	-0.877224
H	3.337683	-4.827645	0.000000
H	-2.557777	6.144813	-0.877224

H	-3.337683	4.827645	0.000000
H	-2.557777	6.144813	0.877224

QDTBDT-Neutral: BS-UB3LYP/6-311G* geometry

C	0.079608	5.288577	0.000000
C	-1.230549	4.718004	0.000000
C	-1.147525	3.331508	0.000000
C	0.152603	2.790822	0.000000
S	1.355333	4.045234	0.000000
C	0.209801	1.387677	0.000000
C	-1.116294	0.827887	0.000000
S	-2.378743	2.074813	0.000000
C	1.332795	0.528482	0.000000
C	1.116294	-0.827887	0.000000
C	-0.209801	-1.387677	0.000000
C	-1.332795	-0.528482	0.000000
C	-0.152603	-2.790822	0.000000
C	1.147525	-3.331508	0.000000
S	2.378743	-2.074813	0.000000
C	1.230549	-4.718004	0.000000
C	-0.079608	-5.288577	0.000000
S	-1.355333	-4.045234	0.000000
C	2.518909	-5.487184	0.000000
C	-0.461770	-6.634442	0.000000
C	-1.836244	-6.989546	0.000000
N	-2.962869	-7.257620	0.000000
C	-2.518909	5.487184	0.000000
C	0.461770	6.634442	0.000000
C	1.836244	6.989546	0.000000
N	2.962869	7.257620	0.000000
C	-0.461770	7.709894	0.000000
N	-1.191007	8.609515	0.000000
C	0.461770	-7.709894	0.000000
N	1.191007	-8.609515	0.000000
H	2.336072	0.940334	0.000000
H	-2.336072	-0.940334	0.000000
H	2.608159	-6.131937	0.877222
H	2.608159	-6.131937	-0.877222
H	3.370273	-4.804755	0.000000
H	-2.608159	6.131937	-0.877222
H	-3.370273	4.804755	0.000000
H	-2.608159	6.131937	0.877222

QDTBDT-Cation: UB3LYP/6-311G* geometry

C	0.115787	5.264127	0.000000
C	-1.213122	4.707214	0.000000
C	-1.138707	3.325178	0.000000
C	0.171133	2.782330	0.000000
S	1.385792	4.011981	0.000000
C	0.216495	1.380044	0.000000
C	-1.118648	0.834342	0.000000
S	-2.376121	2.080998	0.000000
C	1.339963	0.520594	0.000000
C	1.118648	-0.834342	0.000000
C	-0.216495	-1.380044	0.000000
C	-1.339963	-0.520594	0.000000
C	-0.171133	-2.782330	0.000000
C	1.138707	-3.325178	0.000000
S	2.376121	-2.080998	0.000000
C	1.213122	-4.707214	0.000000
C	-0.115787	-5.264127	0.000000
S	-1.385792	-4.011981	0.000000
C	2.488394	-5.489701	0.000000
C	-0.503667	-6.597183	0.000000
C	-1.882752	-6.944473	0.000000
N	-3.010550	-7.201736	0.000000
C	-2.488394	5.489701	0.000000
C	0.503667	6.597183	0.000000
C	1.882752	6.944473	0.000000
N	3.010550	7.201736	0.000000
C	-0.414357	7.679430	0.000000
N	-1.138707	8.581314	0.000000
C	0.414357	-7.679430	0.000000
N	1.138707	-8.581314	0.000000
H	2.344111	0.929844	0.000000
H	-2.344111	-0.929844	0.000000
H	2.565533	-6.136269	0.877576
H	2.565533	-6.136269	-0.877576
H	3.349943	-4.821570	0.000000
H	-2.565533	6.136269	-0.877576
H	-3.349943	4.821570	0.000000
H	-2.565533	6.136269	0.877576

QDTBDT-Anion: UB3LYP/6-311G* geometry

C	0.070343	5.319670	0.000000
C	-1.220867	4.742416	0.000000
C	-1.134221	3.346491	0.000000
C	0.152565	2.803387	0.000000
S	1.346846	4.075618	0.000000

C	0.213413	1.395257	0.000000
C	-1.107296	0.830254	0.000000
S	-2.367378	2.084438	0.000000
C	1.328262	0.527924	0.000000
C	1.107296	-0.830254	0.000000
C	-0.213413	-1.395257	0.000000
C	-1.328262	-0.527924	0.000000
C	-0.152565	-2.803387	0.000000
C	1.134221	-3.346491	0.000000
S	2.367378	-2.084438	0.000000
C	1.220867	-4.742416	0.000000
C	-0.070343	-5.319670	0.000000
S	-1.346846	-4.075618	0.000000
C	2.518169	-5.502797	0.000000
C	-0.460058	-6.681008	0.000000
C	-1.829606	-7.034942	0.000000
N	-2.957521	-7.310652	0.000000
C	-2.518169	5.502797	0.000000
C	0.460058	6.681008	0.000000
C	1.829606	7.034942	0.000000
N	2.957521	7.310652	0.000000
C	-0.460058	7.754233	0.000000
N	-1.188532	8.658445	0.000000
C	0.460058	-7.754233	0.000000
N	1.188532	-8.658445	0.000000
H	2.334373	0.934116	0.000000
H	-2.334373	-0.934116	0.000000
H	2.616769	-6.147819	0.876496
H	2.616769	-6.147819	-0.876496
H	3.363584	-4.811118	0.000000
H	-2.616769	6.147819	-0.876496
H	-3.363584	4.811118	0.000000
H	-2.616769	6.147819	0.876496

TPQ-Neutral: CS-B3LYP/6-311G* geometry

N	-1.530066	10.618678	0.000000
C	-0.711366	9.799057	0.000000
C	0.273999	8.779673	0.000000
C	1.629772	9.194416	0.000000
N	2.745118	9.507870	0.000000
C	-0.072367	7.417516	0.000000
C	-1.453748	7.011163	0.000000
C	-1.816515	5.694111	0.000000
C	-0.810755	4.693092	0.000000
C	0.576954	5.085787	0.000000

C	0.939524	6.396146	0.000000
H	1.983612	6.690337	0.000000
H	-2.216703	7.781070	0.000000
H	-2.866133	5.418867	0.000000
C	-0.866409	3.293652	0.000000
C	0.423000	2.747046	0.000000
C	1.453748	3.841190	0.000000
H	2.107272	3.802168	0.878609
H	2.107272	3.802168	-0.878609
C	-1.896343	2.199958	0.000000
H	-2.549746	2.241960	0.878571
H	-2.549746	2.241960	-0.878571
C	-1.023742	0.953962	0.000000
C	0.367935	1.344835	0.000000
C	1.394438	0.360977	0.000000
H	2.439109	0.656597	0.000000
N	1.530066	-10.618678	0.000000
C	0.711366	-9.799057	0.000000
C	-0.273999	-8.779673	0.000000
C	-1.629772	-9.194416	0.000000
N	-2.745118	-9.507870	0.000000
C	0.072367	-7.417516	0.000000
C	1.453748	-7.011163	0.000000
C	1.816515	-5.694111	0.000000
C	0.810755	-4.693092	0.000000
C	-0.576954	-5.085787	0.000000
C	-0.939524	-6.396146	0.000000
H	-1.983612	-6.690337	0.000000
H	2.216703	-7.781070	0.000000
H	2.866133	-5.418867	0.000000
C	0.866409	-3.293652	0.000000
C	-0.423000	-2.747046	0.000000
C	-1.453748	-3.841190	0.000000
H	-2.107272	-3.802168	-0.878609
H	-2.107272	-3.802168	0.878609
C	1.896343	-2.199958	0.000000
H	2.549746	-2.241960	-0.878571
H	2.549746	-2.241960	0.878571
C	1.023742	-0.953962	0.000000
C	-0.367935	-1.344835	0.000000
C	-1.394438	-0.360977	0.000000
H	-2.439109	-0.656597	0.000000

TPQ-Neutral: BS-UB3LYP/6-311G* geometry

N	-1.519396	10.657430	0.000000

C	-0.702169	9.835342	0.000000
C	0.279293	8.816259	0.000000
C	1.632661	9.228725	0.000000
N	2.748502	9.543073	0.000000
C	-0.074652	7.437699	0.000000
C	-1.445504	7.039493	0.000000
C	-1.810061	5.712167	0.000000
C	-0.808885	4.722379	0.000000
C	0.570180	5.107981	0.000000
C	0.934106	6.425486	0.000000
H	1.979103	6.717237	0.000000
H	-2.211196	7.806913	0.000000
H	-2.859843	5.438092	0.000000
C	-0.859769	3.297110	0.000000
C	0.412962	2.771862	0.000000
C	1.445504	3.863503	0.000000
H	2.099709	3.823902	0.878504
H	2.099709	3.823902	-0.878504
C	-1.888653	2.201910	0.000000
H	-2.542976	2.241638	0.878518
H	-2.542976	2.241638	-0.878518
C	-1.013193	0.957907	0.000000
C	0.364462	1.339712	0.000000
C	1.384850	0.369475	0.000000
H	2.430237	0.663419	0.000000
N	1.519396	-10.657430	0.000000
C	0.702169	-9.835342	0.000000
C	-0.279293	-8.816259	0.000000
C	-1.632661	-9.228725	0.000000
N	-2.748502	-9.543073	0.000000
C	0.074652	-7.437699	0.000000
C	1.445504	-7.039493	0.000000
C	1.810061	-5.712167	0.000000
C	0.808885	-4.722379	0.000000
C	-0.570180	-5.107981	0.000000
C	-0.934106	-6.425486	0.000000
H	-1.979103	-6.717237	0.000000
H	2.211196	-7.806913	0.000000
H	2.859843	-5.438092	0.000000
C	0.859769	-3.297110	0.000000
C	-0.412962	-2.771862	0.000000
C	-1.445504	-3.863503	0.000000
H	-2.099709	-3.823902	-0.878504
H	-2.099709	-3.823902	0.878504
C	1.888653	-2.201910	0.000000
H	2.542976	-2.241638	-0.878518
H	2.542976	-2.241638	0.878518

C	1.013193	-0.957907	0.000000
C	-0.364462	-1.339712	0.000000
C	-1.384850	-0.369475	0.000000
H	-2.430237	-0.663419	0.000000

TPQ-Cation: UB3LYP/6-311G* geometry

N	-1.525363	10.616907	0.000000
C	-0.710173	9.794922	0.000000
C	0.272795	8.773962	0.000000
C	1.627905	9.190184	0.000000
N	2.742132	9.504321	0.000000
C	-0.074974	7.406498	0.000000
C	-1.454695	7.009886	0.000000
C	-1.816581	5.689065	0.000000
C	-0.804521	4.699529	0.000000
C	0.581246	5.082723	0.000000
C	0.943420	6.396612	0.000000
H	1.986580	6.693095	0.000000
H	-2.218467	7.778757	0.000000
H	-2.864240	5.410049	0.000000
C	-0.864026	3.287260	0.000000
C	0.420413	2.752879	0.000000
C	1.454695	3.839457	0.000000
H	2.108098	3.796064	0.878529
H	2.108098	3.796064	-0.878529
C	-1.896085	2.198331	0.000000
H	-2.549651	2.240744	0.878457
H	-2.549651	2.240744	-0.878457
C	-1.023141	0.955257	0.000000
C	0.364963	1.336169	0.000000
C	1.393713	0.365641	0.000000
H	2.437041	0.663681	0.000000
N	1.525363	-10.616907	0.000000
C	0.710173	-9.794922	0.000000
C	-0.272795	-8.773962	0.000000
C	-1.627905	-9.190184	0.000000
N	-2.742132	-9.504321	0.000000
C	0.074974	-7.406498	0.000000
C	1.454695	-7.009886	0.000000
C	1.816581	-5.689065	0.000000
C	0.804521	-4.699529	0.000000
C	-0.581246	-5.082723	0.000000
C	-0.943420	-6.396612	0.000000
H	-1.986580	-6.693095	0.000000
H	2.218467	-7.778757	0.000000

H	2.864240	-5.410049	0.000000
C	0.864026	-3.287260	0.000000
C	-0.420413	-2.752879	0.000000
C	-1.454695	-3.839457	0.000000
H	-2.108098	-3.796064	-0.878529
H	-2.108098	-3.796064	0.878529
C	1.896085	-2.198331	0.000000
H	2.549651	-2.240744	-0.878457
H	2.549651	-2.240744	0.878457
C	1.023141	-0.955257	0.000000
C	-0.364963	-1.336169	0.000000
C	-1.393713	-0.365641	0.000000
H	-2.437041	-0.663681	0.000000

TPQ-Anion: UB3LYP/6-311G* geometry

N	6.643221	8.502344	0.000000
C	6.622644	7.340565	0.000000
C	6.571608	5.929905	0.000000
C	7.802017	5.238369	0.000000
N	8.802928	4.647986	0.000000
C	5.324819	5.227500	0.000000
C	4.084859	5.931198	0.000000
C	2.872261	5.273367	0.000000
C	2.843178	3.868066	0.000000
C	4.078297	3.148369	0.000000
C	5.282818	3.800286	0.000000
H	6.215886	3.245463	0.000000
H	4.105366	7.015569	0.000000
H	1.948709	5.845248	0.000000
C	1.784607	2.910151	0.000000
C	2.286128	1.628898	0.000000
C	3.791270	1.651009	0.000000
H	4.219954	1.154088	0.878468
H	4.219954	1.154088	-0.878468
C	0.280574	2.887166	0.000000
H	-0.145576	3.386294	0.878503
H	-0.145576	3.386294	-0.878503
C	-0.010343	1.392368	0.000000
C	1.224226	0.669994	0.000000
C	1.224226	-0.740486	0.000000
H	2.159965	-1.293079	0.000000
N	-6.643221	-8.502344	0.000000
C	-6.622644	-7.340565	0.000000
C	-6.571608	-5.929905	0.000000
C	-7.802017	-5.238369	0.000000

N	-8.802928	-4.647986	0.000000
C	-5.324819	-5.227500	0.000000
C	-4.084859	-5.931198	0.000000
C	-2.872261	-5.273367	0.000000
C	-2.843178	-3.868066	0.000000
C	-4.078297	-3.148369	0.000000
C	-5.282818	-3.800286	0.000000
H	-6.215886	-3.245463	0.000000
H	-4.105366	-7.015569	0.000000
H	-1.948709	-5.845248	0.000000
C	-1.784607	-2.910151	0.000000
C	-2.286128	-1.628898	0.000000
C	-3.791270	-1.651009	0.000000
H	-4.219954	-1.154088	-0.878468
H	-4.219954	-1.154088	0.878468
C	-0.280574	-2.887166	0.000000
H	0.145576	-3.386294	-0.878503
H	0.145576	-3.386294	0.878503
C	0.010343	-1.392368	0.000000
C	-1.224226	-0.669994	0.000000
C	-1.224226	0.740486	0.000000
H	-2.159965	1.293079	0.000000

NZ-Neutral: CS-B3LYP/6-311G* geometry

C	8.598774	0.669978	0.000000
C	8.641021	-0.706928	0.000000
C	7.442929	-1.471163	0.000000
C	6.183066	-0.796608	0.000000
C	6.161408	0.638261	0.000000
C	7.368157	1.341289	0.000000
H	7.345832	2.426890	0.000000
C	4.903983	1.309686	0.000000
C	3.700516	0.638034	0.000000
C	3.677165	-0.825765	0.000000
C	4.966367	-1.543707	0.000000
H	4.901916	2.396341	0.000000
C	2.462110	1.343896	0.000000
C	1.241136	0.705524	0.000000
C	1.212972	-0.752301	0.000000
C	2.462110	-1.454813	0.000000
H	2.495051	2.430251	0.000000
H	2.404526	-2.537831	0.000000
C	-0.001910	1.406443	0.000000
C	-1.212972	0.752301	0.000000
C	-1.241136	-0.705524	0.000000

C	0.001910	-1.406443	0.000000
H	0.019078	2.493220	0.000000
H	-0.019078	-2.493220	0.000000
C	-2.462110	1.454813	0.000000
C	-3.677165	0.825765	0.000000
C	-3.700516	-0.638034	0.000000
C	-2.462110	-1.343896	0.000000
H	-2.404526	2.537831	0.000000
H	-2.495051	-2.430251	0.000000
C	-4.966367	1.543707	0.000000
C	-6.183066	0.796608	0.000000
C	-6.161408	-0.638261	0.000000
C	-4.903983	-1.309686	0.000000
H	-4.901916	-2.396341	0.000000
C	-7.368157	-1.341289	0.000000
C	-8.598774	-0.669978	0.000000
H	-9.521201	-1.241862	0.000000
C	-8.641021	0.706928	0.000000
H	-9.593388	1.228051	0.000000
C	-7.442929	1.471163	0.000000
H	-7.345832	-2.426890	0.000000
C	-5.052212	2.935209	0.000000
C	-7.465710	2.884937	0.000000
C	-6.287033	3.597599	0.000000
H	-4.151250	3.536700	0.000000
H	-6.306808	4.682742	0.000000
H	-8.422106	3.398852	0.000000
H	9.593388	-1.228051	0.000000
H	9.521201	1.241862	0.000000
C	5.052212	-2.935209	0.000000
C	7.465710	-2.884937	0.000000
C	6.287033	-3.597599	0.000000
H	6.306808	-4.682742	0.000000
H	8.422106	-3.398852	0.000000
H	4.151250	-3.536700	0.000000

NZ-Neutral: BS-UB3LYP/6-311G* geometry

C	8.599021	0.732472	0.000000
C	8.644066	-0.646898	0.000000
C	7.447164	-1.415337	0.000000
C	6.184912	-0.745371	0.000000
C	6.158469	0.692700	0.000000
C	7.370353	1.400744	0.000000
H	7.345006	2.486198	0.000000
C	4.909310	1.357590	0.000000

C	3.691933	0.671587	0.000000
C	3.681605	-0.786252	0.000000
C	4.972153	-1.496493	0.000000
H	4.899160	2.443953	0.000000
C	2.465934	1.367402	0.000000
C	1.232028	0.714289	0.000000
C	1.219672	-0.736297	0.000000
C	2.465934	-1.428519	0.000000
H	2.488448	2.453899	0.000000
H	2.418709	-2.511832	0.000000
C	-0.001541	1.404854	0.000000
C	-1.219672	0.736297	0.000000
C	-1.232028	-0.714289	0.000000
C	0.001541	-1.404854	0.000000
H	0.008837	2.491711	0.000000
H	-0.008837	-2.491711	0.000000
C	-2.465934	1.428519	0.000000
C	-3.681605	0.786252	0.000000
C	-3.691933	-0.671587	0.000000
C	-2.465934	-1.367402	0.000000
H	-2.418709	2.511832	0.000000
H	-2.488448	-2.453899	0.000000
C	-4.972153	1.496493	0.000000
C	-6.184912	0.745371	0.000000
C	-6.158469	-0.692700	0.000000
C	-4.909310	-1.357590	0.000000
H	-4.899160	-2.443953	0.000000
C	-7.370353	-1.400744	0.000000
C	-8.599021	-0.732472	0.000000
H	-9.520897	-1.305344	0.000000
C	-8.644066	0.646898	0.000000
H	-9.597639	1.165749	0.000000
C	-7.447164	1.415337	0.000000
H	-7.345006	-2.486198	0.000000
C	-5.062860	2.890422	0.000000
C	-7.475718	2.827977	0.000000
C	-6.298783	3.546692	0.000000
H	-4.164163	3.495052	0.000000
H	-6.323737	4.631759	0.000000
H	-8.434143	3.338116	0.000000
H	9.597639	-1.165749	0.000000
H	9.520897	1.305344	0.000000
C	5.062860	-2.890422	0.000000
C	7.475718	-2.827977	0.000000
C	6.298783	-3.546692	0.000000
H	6.323737	-4.631759	0.000000
H	8.434143	-3.338116	0.000000

H	4.164163	-3.495052	0.000000

NZ-Cation: UB3LYP/6-311G* geometry

C	8.574138	0.759058	0.000000
C	8.622136	-0.623565	0.000000
C	7.437146	-1.405654	0.000000
C	6.170659	-0.749306	0.000000
C	6.137762	0.686383	0.000000
C	7.341560	1.412293	0.000000
H	7.303690	2.496826	0.000000
C	4.890881	1.343880	0.000000
C	3.678779	0.650597	0.000000
C	3.674520	-0.808583	0.000000
C	4.968230	-1.508517	0.000000
H	4.873869	2.429822	0.000000
C	2.459962	1.356581	0.000000
C	1.228115	0.705394	0.000000
C	1.217540	-0.747404	0.000000
C	2.459962	-1.448425	0.000000
H	2.488180	2.442092	0.000000
H	2.407993	-2.530875	0.000000
C	0.001232	1.408647	0.000000
C	-1.217540	0.747404	0.000000
C	-1.228115	-0.705394	0.000000
C	-0.001232	-1.408647	0.000000
H	0.021905	2.494404	0.000000
H	-0.021905	-2.494404	0.000000
C	-2.459962	1.448425	0.000000
C	-3.674520	0.808583	0.000000
C	-3.678779	-0.650597	0.000000
C	-2.459962	-1.356581	0.000000
H	-2.407993	2.530875	0.000000
H	-2.488180	-2.442092	0.000000
C	-4.968230	1.508517	0.000000
C	-6.170659	0.749306	0.000000
C	-6.137762	-0.686383	0.000000
C	-4.890881	-1.343880	0.000000
H	-4.873869	-2.429822	0.000000
C	-7.341560	-1.412293	0.000000
C	-8.574138	-0.759058	0.000000
H	-9.492055	-1.335440	0.000000
C	-8.622136	0.623565	0.000000
H	-9.580768	1.132275	0.000000

C	-7.437146	1.405654	0.000000
H	-7.303690	-2.496826	0.000000
C	-5.074167	2.901941	0.000000
C	-7.484946	2.817698	0.000000
C	-6.315139	3.548626	0.000000
H	-4.182202	3.516338	0.000000
H	-6.349900	4.632267	0.000000
H	-8.447706	3.317719	0.000000
H	9.580768	-1.132275	0.000000
H	9.492055	1.335440	0.000000
C	5.074167	-2.901941	0.000000
C	7.484946	-2.817698	0.000000
C	6.315139	-3.548626	0.000000
H	6.349900	-4.632267	0.000000
H	8.447706	-3.317719	0.000000
H	4.182202	-3.516338	0.000000

NZ-Anion: UB3LYP/6-311G* geometry

C	8.615988	0.708740	0.000000
C	8.660852	-0.673521	0.000000
C	7.456398	-1.428583	0.000000
C	6.194432	-0.747468	0.000000
C	6.170233	0.696699	0.000000
C	7.397682	1.389326	0.000000
H	7.383698	2.476008	0.000000
C	4.927201	1.364289	0.000000
C	3.703969	0.688576	0.000000
C	3.686573	-0.772198	0.000000
C	4.975453	-1.491049	0.000000
H	4.922852	2.451819	0.000000
C	2.471095	1.374266	0.000000
C	1.237615	0.723321	0.000000
C	1.219488	-0.730259	0.000000
C	2.471095	-1.415173	0.000000
H	2.489154	2.462210	0.000000
H	2.426610	-2.499390	0.000000
C	-0.004536	1.402419	0.000000
C	-1.219488	0.730259	0.000000
C	-1.237615	-0.723321	0.000000
C	0.004536	-1.402419	0.000000
H	-0.002519	2.490629	0.000000
H	0.002519	-2.490629	0.000000
C	-2.471095	1.415173	0.000000
C	-3.686573	0.772198	0.000000
C	-3.703969	-0.688576	0.000000

C	-2.471095	-1.374266	0.000000
H	-2.426610	2.499390	0.000000
H	-2.489154	-2.462210	0.000000
C	-4.975453	1.491049	0.000000
C	-6.194432	0.747468	0.000000
C	-6.170233	-0.696699	0.000000
C	-4.927201	-1.364289	0.000000
H	-4.922852	-2.451819	0.000000
C	-7.397682	-1.389326	0.000000
C	-8.615988	-0.708740	0.000000
H	-9.543064	-1.277431	0.000000
C	-8.660852	0.673521	0.000000
H	-9.611566	1.199222	0.000000
C	-7.456398	1.428583	0.000000
H	-7.383698	-2.476008	0.000000
C	-5.052806	2.888603	0.000000
C	-7.467398	2.843156	0.000000
C	-6.283771	3.551821	0.000000
H	-4.148171	3.484420	0.000000
H	-6.301320	4.638664	0.000000
H	-8.422314	3.362502	0.000000
H	9.611566	-1.199222	0.000000
H	9.543064	1.277431	0.000000
C	5.052806	-2.888603	0.000000
C	7.467398	-2.843156	0.000000
C	6.283771	-3.551821	0.000000
H	6.301320	-4.638664	0.000000
H	8.422314	-3.362502	0.000000
H	4.148171	-3.484420	0.000000

Ph_2-IDPL-Neutral: CS-B3LYP/6-311G* geometry

C	-1.109102	0.141698	-0.921942
C	-0.943763	-1.026085	-0.167205
C	0.168041	-1.169681	0.757752
C	0.065698	-2.471067	1.380480
C	0.794772	-3.218791	2.339659
C	0.366259	-4.484461	2.694947
C	-0.802930	-5.099450	2.133790
C	-1.280545	-6.378733	2.459798
C	-2.435981	-6.904165	1.861365
C	-3.136323	-6.144570	0.919014
C	-2.708517	-4.854934	0.549458
C	-3.362973	-4.009029	-0.404042
C	-2.900210	-2.746124	-0.731850
C	-1.730672	-2.242780	-0.111899

C	-1.087597	-3.080004	0.828832
C	-1.535825	-4.352441	1.172070
C	-2.251297	0.289327	-1.872751
C	-3.466891	0.834093	-1.444718
C	-4.532407	0.972143	-2.332101
C	-4.396015	0.567246	-3.658882
C	-3.188425	0.023474	-4.093512
C	-2.122892	-0.114620	-3.206140
C	-2.907761	-8.290653	2.234996
H	1.686259	-2.812931	2.801330
H	0.936197	-5.044016	3.431512
H	-0.744481	-6.977278	3.192632
H	-4.031115	-6.559086	0.461799
H	-4.261753	-4.380470	-0.888466
H	-3.441839	-2.157186	-1.461577
H	-3.574197	1.149616	-0.412104
H	-5.469609	1.396419	-1.985864
H	-5.225915	0.674718	-4.350034
H	-3.074308	-0.294352	-5.125109
H	-1.183281	-0.538046	-3.545548
H	-3.029952	-8.394272	3.317384
H	-3.866811	-8.527504	1.770033
H	-2.190734	-9.055060	1.918009
C	1.109102	-0.141698	0.921942
C	0.943763	1.026085	0.167205
C	-0.168041	1.169681	-0.757752
C	-0.065698	2.471067	-1.380480
C	-0.794772	3.218791	-2.339659
C	-0.366259	4.484461	-2.694947
C	0.802930	5.099450	-2.133790
C	1.280545	6.378733	-2.459798
C	2.435981	6.904165	-1.861365
C	3.136323	6.144570	-0.919014
C	2.708517	4.854934	-0.549458
C	3.362973	4.009029	0.404042
C	2.900210	2.746124	0.731850
C	1.730672	2.242780	0.111899
C	1.087597	3.080004	-0.828832
C	1.535825	4.352441	-1.172070
C	2.251297	-0.289327	1.872751
C	3.466891	-0.834093	1.444718
C	4.532407	-0.972143	2.332101
C	4.396015	-0.567246	3.658882
C	3.188425	-0.023474	4.093512
C	2.122892	0.114620	3.206140
C	2.907761	8.290653	-2.234996
H	-1.686259	2.812931	-2.801330

H	-0.936197	5.044016	-3.431512
H	0.744481	6.977278	-3.192632
H	4.031115	6.559086	-0.461799
H	4.261753	4.380470	0.888466
H	3.441839	2.157186	1.461577
H	3.574197	-1.149616	0.412104
H	5.469609	-1.396419	1.985864
H	5.225915	-0.674718	4.350034
H	3.074308	0.294352	5.125109
H	1.183281	0.538046	3.545548
H	3.029952	8.394272	-3.317384
H	3.866811	8.527504	-1.770033
H	2.190734	9.055060	-1.918009

Ph_2-IDPL-Neutral: BS-UB3LYP/6-311G* geometry

C	-1.107512	0.141774	-0.920764
C	-0.940383	-1.025057	-0.165091
C	0.166401	-1.167737	0.755540
C	0.063620	-2.478100	1.382754
C	0.791462	-3.220672	2.338241
C	0.362851	-4.491215	2.696073
C	-0.803871	-5.104390	2.135854
C	-1.283047	-6.386048	2.461964
C	-2.438545	-6.910623	1.862990
C	-3.138899	-6.151124	0.920675
C	-2.710399	-4.859291	0.550464
C	-3.363521	-4.015032	-0.401188
C	-2.897543	-2.748081	-0.728703
C	-1.732092	-2.249088	-0.109516
C	-1.088880	-3.085404	0.830843
C	-1.537227	-4.357481	1.173825
C	-2.250277	0.289082	-1.871850
C	-3.466496	0.833341	-1.444447
C	-4.532271	0.971190	-2.331608
C	-4.395864	0.566453	-3.658432
C	-3.188068	0.023160	-4.093107
C	-2.122579	-0.114526	-3.205591
C	-2.910641	-8.297007	2.236234
H	1.683009	-2.814175	2.799598
H	0.933709	-5.049575	3.432728
H	-0.747174	-6.984726	3.194721
H	-4.033714	-6.565262	0.463265
H	-4.262382	-4.384946	-0.886486
H	-3.438596	-2.158393	-1.458441

H	-3.574100	1.148861	-0.411801
H	-5.469609	1.395164	-1.985313
H	-5.225872	0.673722	-4.349529
H	-3.073888	-0.294512	-5.124766
H	-1.182864	-0.537633	-3.545299
H	-3.031826	-8.401074	3.318666
H	-3.870244	-8.533208	1.771999
H	-2.194108	-9.061412	1.918285
C	1.107512	-0.141774	0.920764
C	0.940383	1.025057	0.165091
C	-0.166401	1.167737	-0.755540
C	-0.063620	2.478100	-1.382754
C	-0.791462	3.220672	-2.338241
C	-0.362851	4.491215	-2.696073
C	0.803871	5.104390	-2.135854
C	1.283047	6.386048	-2.461964
C	2.438545	6.910623	-1.862990
C	3.138899	6.151124	-0.920675
C	2.710399	4.859291	-0.550464
C	3.363521	4.015032	0.401188
C	2.897543	2.748081	0.728703
C	1.732092	2.249088	0.109516
C	1.088880	3.085404	-0.830843
C	1.537227	4.357481	-1.173825
C	2.250277	-0.289082	1.871850
C	3.466496	-0.833341	1.444447
C	4.532271	-0.971190	2.331608
C	4.395864	-0.566453	3.658432
C	3.188068	-0.023160	4.093107
C	2.122579	0.114526	3.205591
C	2.910641	8.297007	-2.236234
H	-1.683009	2.814175	-2.799598
H	-0.933709	5.049575	-3.432728
H	0.747174	6.984726	-3.194721
H	4.033714	6.565262	-0.463265
H	4.262382	4.384946	0.886486
H	3.438596	2.158393	1.458441
H	3.574100	-1.148861	0.411801
H	5.469609	-1.395164	1.985313
H	5.225872	-0.673722	4.349529
H	3.073888	0.294512	5.124766
H	1.182864	0.537633	3.545299
H	3.031826	8.401074	-3.318666
H	3.870244	8.533208	-1.771999
H	2.194108	9.061412	-1.918285

Ph$_2$-IDPL-Cation: UB3LYP/6-311G* geometry

C	-1.110363	0.142582	-0.923346
C	-0.935095	-1.021287	-0.163081
C	0.163655	-1.162969	0.750839
C	0.060574	-2.482816	1.382798
C	0.790446	-3.216647	2.334916
C	0.357569	-4.492498	2.692240
C	-0.805399	-5.101016	2.132483
C	-1.284852	-6.385581	2.459696
C	-2.440154	-6.915370	1.863755
C	-3.137344	-6.151004	0.921416
C	-2.707291	-4.856924	0.551373
C	-3.359726	-4.017016	-0.397326
C	-2.894084	-2.744127	-0.728204
C	-1.732815	-2.253301	-0.107825
C	-1.089973	-3.087937	0.831242
C	-1.537207	-4.356812	1.173322
C	-2.252411	0.289974	-1.873952
C	-3.467135	0.834800	-1.442283
C	-4.532130	0.972117	-2.330792
C	-4.394124	0.566979	-3.657479
C	-3.186806	0.023448	-4.093640
C	-2.120317	-0.114953	-3.207107
C	-2.911247	-8.299923	2.238365
H	1.681268	-2.810727	2.795959
H	0.929185	-5.049212	3.428478
H	-0.748194	-6.982712	3.192083
H	-4.031794	-6.563450	0.463553
H	-4.258150	-4.385028	-0.883241
H	-3.435013	-2.155249	-1.457303
H	-3.577549	1.151047	-0.410084
H	-5.469332	1.395730	-1.985529
H	-5.223613	0.674232	-4.348216
H	-3.073496	-0.293760	-5.124920
H	-1.181661	-0.538448	-3.549465
H	-3.031175	-8.400062	3.320332
H	-3.869527	-8.538341	1.774970
H	-2.192161	-9.060694	1.920611
C	1.110363	-0.142582	0.923346
C	0.935095	1.021287	0.163081
C	-0.163655	1.162969	-0.750839
C	-0.060574	2.482816	-1.382798
C	-0.790446	3.216647	-2.334916
C	-0.357569	4.492498	-2.692240
C	0.805399	5.101016	-2.132483

C	1.284852	6.385581	-2.459696
C	2.440154	6.915370	-1.863755
C	3.137344	6.151004	-0.921416
C	2.707291	4.856924	-0.551373
C	3.359726	4.017016	0.397326
C	2.894084	2.744127	0.728204
C	1.732815	2.253301	0.107825
C	1.089973	3.087937	-0.831242
C	1.537207	4.356812	-1.173322
C	2.252411	-0.289974	1.873952
C	3.467135	-0.834800	1.442283
C	4.532130	-0.972117	2.330792
C	4.394124	-0.566979	3.657479
C	3.186806	-0.023448	4.093640
C	2.120317	0.114953	3.207107
C	2.911247	8.299923	-2.238365
H	-1.681268	2.810727	-2.795959
H	-0.929185	5.049212	-3.428478
H	0.748194	6.982712	-3.192083
H	4.031794	6.563450	-0.463553
H	4.258150	4.385028	0.883241
H	3.435013	2.155249	1.457303
H	3.577549	-1.151047	0.410084
H	5.469332	-1.395730	1.985529
H	5.223613	-0.674232	4.348216
H	3.073496	0.293760	5.124920
H	1.181661	0.538448	3.549465
H	3.031175	8.400062	-3.320332
H	3.869527	8.538341	-1.774970
H	2.192161	9.060694	-1.920611

Ph_2-IDPL-Anion: UB3LYP/6-311G* geometry

C	-1.101832	0.139158	-0.922149
C	-0.944398	-1.032318	-0.166767
C	0.166698	-1.172663	0.763104
C	0.062883	-2.477716	1.390929
C	0.782512	-3.223626	2.351021
C	0.356019	-4.493238	2.713501
C	-0.807459	-5.109686	2.150479
C	-1.291525	-6.392143	2.478973
C	-2.442431	-6.912403	1.875201
C	-3.144959	-6.158094	0.927978
C	-2.715861	-4.868683	0.553424
C	-3.366443	-4.023139	-0.401798
C	-2.896544	-2.759008	-0.728039

C	-1.731625	-2.251064	-0.110811
C	-1.089133	-3.087613	0.835321
C	-1.538981	-4.362765	1.180404
C	-2.241948	0.283222	-1.876759
C	-3.461193	0.829179	-1.459313
C	-4.525443	0.964504	-2.348699
C	-4.387497	0.554942	-3.673949
C	-3.178035	0.009648	-4.101643
C	-2.115743	-0.124368	-3.209734
C	-2.909859	-8.308892	2.223204
H	1.673246	-2.816960	2.816700
H	0.925167	-5.049359	3.454879
H	-0.759379	-6.989246	3.217104
H	-4.040641	-6.574887	0.471257
H	-4.265323	-4.393850	-0.889061
H	-3.437936	-2.171589	-1.461190
H	-3.568735	1.148178	-0.427768
H	-5.463863	1.390707	-2.005496
H	-5.216312	0.659908	-4.368190
H	-3.060380	-0.312559	-5.132373
H	-1.174303	-0.548820	-3.542807
H	-2.645237	-8.577552	3.250105
H	-3.994903	-8.406588	2.123792
H	-2.456950	-9.061586	1.566189
C	1.101832	-0.139158	0.922149
C	0.944398	1.032318	0.166767
C	-0.166698	1.172663	-0.763104
C	-0.062883	2.477716	-1.390929
C	-0.782512	3.223626	-2.351021
C	-0.356019	4.493238	-2.713501
C	0.807459	5.109686	-2.150479
C	1.291525	6.392143	-2.478973
C	2.442431	6.912403	-1.875201
C	3.144959	6.158094	-0.927978
C	2.715861	4.868683	-0.553424
C	3.366443	4.023139	0.401798
C	2.896544	2.759008	0.728039
C	1.731625	2.251064	0.110811
C	1.089133	3.087613	-0.835321
C	1.538981	4.362765	-1.180404
C	2.241948	-0.283222	1.876759
C	3.461193	-0.829179	1.459313
C	4.525443	-0.964504	2.348699
C	4.387497	-0.554942	3.673949
C	3.178035	-0.009648	4.101643
C	2.115743	0.124368	3.209734
C	2.909859	8.308892	-2.223204

H	-1.673246	2.816960	-2.816700
H	-0.925167	5.049359	-3.454879
H	0.759379	6.989246	-3.217104
H	4.040641	6.574887	-0.471257
H	4.265323	4.393850	0.889061
H	3.437936	2.171589	1.461190
H	3.568735	-1.148178	0.427768
H	5.463863	-1.390707	2.005496
H	5.216312	-0.659908	4.368190
H	3.060380	0.312559	5.132373
H	1.174303	0.548820	3.542807
H	2.645237	8.577552	-3.250105
H	3.994903	8.406588	-2.123792
H	2.456950	9.061586	-1.566189

BISPHE-Neutral: CS-B3LYP/6-311G* geometry

C	1.322393	5.672938	-6.525590
C	1.935339	5.902888	-5.292747
C	0.504555	4.563645	-6.747064
C	1.741090	5.007428	-4.224403
C	0.273563	3.633755	-5.716077
C	2.318712	5.142270	-2.917079
C	0.905058	3.881535	-4.465288
C	-0.546063	2.460577	-5.829018
C	2.093467	4.229929	-1.901546
C	0.694467	2.977755	-3.425785
C	-0.738955	1.578483	-4.780615
C	1.263912	3.111101	-2.137707
C	-0.113343	1.821863	-3.537600
C	0.796038	1.976069	-1.376515
C	-0.061232	1.173857	-2.247608
C	1.018006	1.577262	-0.074386
C	-0.645221	0.020926	-1.764369
C	0.424829	0.397504	0.431640
H	2.565063	6.777332	-5.157991
H	0.044569	4.418024	-7.719982
H	2.957947	5.999350	-2.726011
H	-1.031285	2.265035	-6.780880
H	2.558677	4.386113	-0.932927
H	-1.370843	0.707687	-4.927166
C	-1.322393	-5.672938	6.525590
C	-1.935339	-5.902888	5.292747
C	-0.504555	-4.563645	6.747064
C	-1.741090	-5.007428	4.224403

C	-0.273563	-3.633755	5.716077
C	-2.318712	-5.142270	2.917079
C	-0.905058	-3.881535	4.465288
C	0.546063	-2.460577	5.829018
C	-2.093467	-4.229929	1.901546
C	-0.694467	-2.977755	3.425785
C	0.738955	-1.578483	4.780615
C	-1.263912	-3.111101	2.137707
C	0.113343	-1.821863	3.537600
C	-0.796038	-1.976069	1.376515
C	0.061232	-1.173857	2.247608
C	-1.018006	-1.577262	0.074386
C	0.645221	-0.020926	1.764369
C	-0.424829	-0.397504	-0.431640
H	-2.565063	-6.777332	5.157991
H	-0.044569	-4.418024	7.719982
H	-2.957947	-5.999350	2.726011
H	1.031285	-2.265035	6.780880
H	-2.558677	-4.386113	0.932927
H	1.370843	-0.707687	4.927166
H	1.486415	6.376987	-7.335297
H	-1.486415	-6.376987	7.335297
H	1.284695	0.584471	2.401831
H	-1.653287	-2.164886	-0.583567
H	-1.284695	-0.584471	-2.401831
H	1.653287	2.164886	0.583567

BISPHE-Neutral: BS-UB3LYP/6-311G* geometry

C	1.324049	5.680098	-6.533827
C	1.937146	5.910339	-5.301140
C	0.506165	4.570921	-6.755615
C	1.742685	5.013088	-4.230234
C	0.274479	3.638799	-5.722557
C	2.318816	5.147750	-2.926601
C	0.906339	3.886937	-4.471508
C	-0.542942	2.468926	-5.835389
C	2.091292	4.230096	-1.907754
C	0.695851	2.983532	-3.432431
C	-0.735430	1.584032	-4.780951
C	1.265350	3.117604	-2.145486
C	-0.111632	1.828641	-3.545075
C	0.793921	1.972932	-1.376601
C	-0.059745	1.174019	-2.244100
C	1.017767	1.578992	-0.077959
C	-0.643797	0.024048	-1.766398

C	0.422953	0.395795	0.429777
H	2.566869	6.784680	-5.166194
H	0.046101	4.425133	-7.728404
H	2.958309	6.004170	-2.733662
H	-1.028965	2.271735	-6.786457
H	2.556237	4.385704	-0.938825
H	-1.367240	0.712966	-4.926822
C	-1.324049	-5.680098	6.533827
C	-1.937146	-5.910339	5.301140
C	-0.506165	-4.570921	6.755615
C	-1.742685	-5.013088	4.230234
C	-0.274479	-3.638799	5.722557
C	-2.318816	-5.147750	2.926601
C	-0.906339	-3.886937	4.471508
C	0.542942	-2.468926	5.835389
C	-2.091292	-4.230096	1.907754
C	-0.695851	-2.983532	3.432431
C	0.735430	-1.584032	4.780951
C	-1.265350	-3.117604	2.145486
C	0.111632	-1.828641	3.545075
C	-0.793921	-1.972932	1.376601
C	0.059745	-1.174019	2.244100
C	-1.017767	-1.578992	0.077959
C	0.643797	-0.024048	1.766398
C	-0.422953	-0.395795	-0.429777
H	-2.566869	-6.784680	5.166194
H	-0.046101	-4.425133	7.728404
H	-2.958309	-6.004170	2.733662
H	1.028965	-2.271735	6.786457
H	-2.556237	-4.385704	0.938825
H	1.367240	-0.712966	4.926822
H	1.488050	6.384130	-7.343519
H	-1.488050	-6.384130	7.343519
H	1.283009	0.580569	2.404576
H	-1.653091	-2.167228	-0.579129
H	-1.283009	-0.580569	-2.404576
H	1.653091	2.167228	0.579129

BISPHE-Cation: UB3LYP/6-311G* geometry

C	1.323402	5.677307	-6.530619
C	1.935038	5.904543	-5.296519
C	0.506002	4.566959	-6.749012
C	1.739392	5.005788	-4.226119
C	0.275261	3.635333	-5.714287
C	2.315476	5.142153	-2.925651

C	0.905462	3.883187	-4.467193
C	-0.541071	2.468246	-5.829115
C	2.090501	4.225832	-1.902336
C	0.695497	2.982046	-3.430718
C	-0.736741	1.579315	-4.776037
C	1.264528	3.116232	-2.145231
C	-0.111192	1.828457	-3.543533
C	0.791705	1.968807	-1.375141
C	-0.058774	1.172825	-2.239446
C	1.018443	1.579252	-0.076481
C	-0.644728	0.022707	-1.766636
C	0.421805	0.394748	0.428634
H	2.564395	6.778324	-5.161585
H	0.046181	4.421200	-7.721179
H	2.954661	5.997960	-2.732477
H	-1.026968	2.270860	-6.779496
H	2.555906	4.383247	-0.935384
H	-1.368185	0.709963	-4.923988
C	-1.323402	-5.677307	6.530619
C	-1.935038	-5.904543	5.296519
C	-0.506002	-4.566959	6.749012
C	-1.739392	-5.005788	4.226119
C	-0.275261	-3.635333	5.714287
C	-2.315476	-5.142153	2.925651
C	-0.905462	-3.883187	4.467193
C	0.541071	-2.468246	5.829115
C	-2.090501	-4.225832	1.902336
C	-0.695497	-2.982046	3.430718
C	0.736741	-1.579315	4.776037
C	-1.264528	-3.116232	2.145231
C	0.111192	-1.828457	3.543533
C	-0.791705	-1.968807	1.375141
C	0.058774	-1.172825	2.239446
C	-1.018443	-1.579252	0.076481
C	0.644728	-0.022707	1.766636
C	-0.421805	-0.394748	-0.428634
H	-2.564395	-6.778324	5.161585
H	-0.046181	-4.421200	7.721179
H	-2.954661	-5.997960	2.732477
H	1.026968	-2.270860	6.779496
H	-2.555906	-4.383247	0.935384
H	1.368185	-0.709963	4.923988
H	1.487183	6.380374	-7.339202
H	-1.487183	-6.380374	7.339202
H	1.283253	0.580691	2.405187
H	-1.653434	-2.167794	-0.579227
H	-1.283253	-0.580691	-2.405187

H	1.653434	2.167794	0.579227

BISPHE-Anion: UB3LYP/6-311G* geometry

C	1.325018	5.684219	-6.538575
C	1.939602	5.917360	-5.307069
C	0.506548	4.576017	-6.763637
C	1.746338	5.021271	-4.234998
C	0.273672	3.642826	-5.731838
C	2.322646	5.154405	-2.928196
C	0.907272	3.890946	-4.476118
C	-0.544937	2.470168	-5.842872
C	2.092974	4.236159	-1.914012
C	0.696161	2.984882	-3.433980
C	-0.734456	1.589468	-4.787900
C	1.266419	3.118716	-2.144585
C	-0.112654	1.827810	-3.546287
C	0.797737	1.981068	-1.380734
C	-0.060872	1.177481	-2.253297
C	1.017372	1.578661	-0.078317
C	-0.643403	0.024369	-1.766031
C	0.425221	0.397942	0.432103
H	2.569958	6.792844	-5.172467
H	0.046183	4.430504	-7.737715
H	2.962816	6.011919	-2.735372
H	-1.031368	2.273047	-6.795164
H	2.558367	4.391508	-0.943396
H	-1.367130	0.716902	-4.933432
C	-1.325018	-5.684219	6.538575
C	-1.939602	-5.917360	5.307069
C	-0.506548	-4.576017	6.763637
C	-1.746338	-5.021271	4.234998
C	-0.273672	-3.642826	5.731838
C	-2.322646	-5.154405	2.928196
C	-0.907272	-3.890946	4.476118
C	0.544937	-2.470168	5.842872
C	-2.092974	-4.236159	1.914012
C	-0.696161	-2.984882	3.433980
C	0.734456	-1.589468	4.787900
C	-1.266419	-3.118716	2.144585
C	0.112654	-1.827810	3.546287
C	-0.797737	-1.981068	1.380734
C	0.060872	-1.177481	2.253297
C	-1.017372	-1.578661	0.078317
C	0.643403	-0.024369	1.766031
C	-0.425221	-0.397942	-0.432103

H	-2.569958	-6.792844	5.172467
H	-0.046183	-4.430504	7.737715
H	-2.962816	-6.011919	2.735372
H	1.031368	-2.273047	6.795164
H	-2.558367	-4.391508	0.943396
H	1.367130	-0.716902	4.933432
H	1.489343	6.389623	-7.349847
H	-1.489343	-6.389623	7.349847
H	1.283579	0.582039	2.403649
H	-1.653141	-2.166450	-0.580782
H	-1.283579	-0.582039	-2.403649
H	1.653141	2.166450	0.580782
